# Advancements in Surfactant Carriers for Enhanced Oil Recovery: Mechanisms, Challenges, and Opportunities

**DOI:** 10.1021/acsomega.4c04058

**Published:** 2024-07-22

**Authors:** Kelly C. B. Maia, Agatha Densy dos Santos Francisco, Mateus Perissé Moreira, Regina S. V. Nascimento, Daniel Grasseschi

**Affiliations:** † Instituto de Química, Universidade Federal do Rio de Janeiro (UFRJ), 21941-909 Rio de Janeiro, Brazil

## Abstract

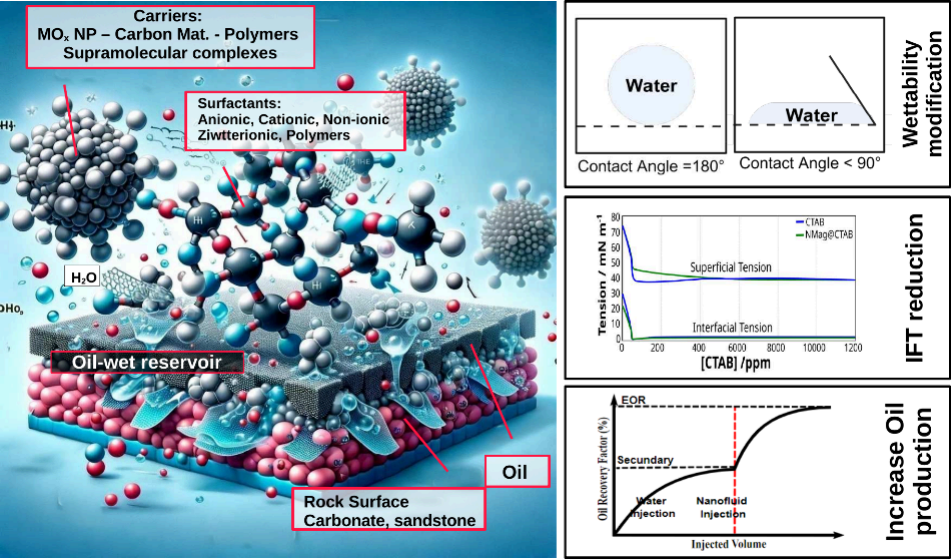

Enhanced oil recovery (EOR) techniques are crucial for maximizing the extraction of residual oil from mature reservoirs. This review explores the latest advancements in surfactant carriers for EOR, focusing on their mechanisms, challenges, and opportunities. We delve into the role of inorganic nanoparticles, carbon materials, polymers and polymeric surfactants, and supramolecular systems, highlighting their interactions with reservoir rocks and their potential to improve oil recovery rates. The discussion includes the formulation and behavior of nanofluids, the impact of surfactant adsorption on different rock types, and innovative approaches using environmentally friendly materials. Notably, the use of metal oxide nanoparticles, carbon nanotubes, graphene derivatives, and polymeric surfacants and the development of supramolecular complexes for managing surfacant delivery are examined. We address the need for further research to optimize these technologies and overcome current limitations, emphasizing the importance of sustainable and economically viable EOR methods. This review aims to provide a comprehensive understanding of the emerging trends and future directions in surfactant carriers for EOR.

## 1 Introduction

Fossil fuels still supply 33% of the world’s energy despite investments in renewable sources, and global energy demand is expected to increase by 38% between 2023 and 2035 due to economic expansion and population growth in emerging countries. To meet this demand, oil production needs to increase by 15%, making the increase of production from oil fields a crucial objective for the oil and gas industry.^1^

In 2016, the average expected recovery factor for hydrocarbon deposits in Brazil ranged from 15% to 20%, while the global average was between 30 and 35%. This indicates that 80–70% of the existing oil remains trapped in the reservoir,^2^ necessitating the development of advanced oil recovery methods, such as enhanced oil recovery (EOR). These technologies can improve production from mature or new fields, such as the Brazilian Presalt, where carbonate reservoirs are responsible for 71.2% of Brazilian oil production. Even small increments in oil recovery can significantly increase oil revenues.^2^

EOR methods aim to obtain more oil than primary and secondary recovery methods, encompassing natural production and water injection. These methods are considered conventional, while tertiary recovery has been renamed EOR.^3,4^ The three methods can be applied chronologically, or not, to increase the recovery factor.^4−6^ In this context, chemical EOR methods aim to increase the oil recovery factor by improving displacement and/or sweeping efficiency. One of the most commonly used EOR methods is the injection of surfactants, polymers, alkalis, or any combination thereof, known as ASP.^7,7−15^ Currently, studies on foam injection,^16−21^ low salinity water,^22−28^ and the use of nanoparticles^29−33^ are gaining momentum in the literature. Each system has a unique mechanism to increase the oil recovery factor, and the selection of the appropriate method for application depends on several factors, such as reservoir type, stability, and cost, among others.

Surfactant injection is a widely used chemical EOR method that aims to change the rocks’ wettability, reduce the interfacial tension between the oil and water phases, making the oil more mobile and easier to displace from the reservoir rock. This method has been successfully applied in several field projects, and recent studies have focused on optimizing the formulation of surfactant solutions and understanding the mechanisms of oil displacement by surfactants.^7,8^ Despite their high efficiency, the surfactants used in EOR processes must be carefully evaluated due to their production cost, toxicity, and tendency to adsorb on the reservoir surfaces.^34^

In recent years, the development of carrier systems for the efficient and targeted delivery of surfactants has garnered significant attention, particularly for applications in EOR. Among the various types of carrier systems, those utilizing supramolecular technologies have stood out due to their unique self-assembly properties and ability to form complex, functional structures. However, it is essential to recognize the broad spectrum of delivery approaches, encompassing both supramolecular and nonsupramolecular systems, each with distinct advantages and specific applications.^35−40^

Supramolecular carrier systems exploit noncovalent interactions, such as hydrogen bonding, van der Waals forces, hydrophobic interactions, pi-pi stacking, and ion-dipole interactions, to create highly ordered structures capable of encapsulating and releasing surfactants in a controlled manner.^41−43^ The self-assembly of these systems enables the formation of micelles, vesicles, and other nanostructures with tunable characteristics, designed to respond to external stimuli such as changes in pH, temperature, or the presence of specific ions. This responsiveness allows for the controlled and localized release of surfactants, enhancing the efficiency of the EOR process.

On the other hand, nonsupramolecular carrier systems, including inorganic or polymer nanoparticles, liposomes, and other nanostructured materials, such as carbon nanotubes and graphene, have also demonstrated promising results.^44,45^ These systems often utilize covalent bonding and physical encapsulation methods to protect and transport active surfactant molecules.^21,46,47^ Surface engineering of these materials can be tailored to improve surfactant transport and release, making them suitable for a wide range of industrial applications, including EOR.

Biocompatibility and biodegradability are crucial aspects for both supramolecular and nonsupramolecular carrier systems.^21^ The use of biocompatible components minimizes toxicity and adverse effects, ensuring the safety of these systems for environmental applications.^3,7,48^ Furthermore, the ability to degrade into nontoxic byproducts after releasing the surfactant reduces environmental impact and facilitates disposal.

In the context of EOR, advanced carrier systems play a significant role in enhancing oil recovery from mature reservoirs. Both supramolecular and nonsupramolecular systems can alter the interfacial properties between oil and water, reducing interfacial tension (IFT) and increasing oil mobility.^46,49,50^ These carrier systems can be engineered to deliver surfactants and other chemical agents in a controlled manner, optimizing the recovery process by responding to specific reservoir conditions such as temperature and salinity variations, thereby improving efficiency and reducing operational costs. Therefore, experimental and theoretical studies on the carrier-surfactant interaction are fundamental to help screen the systems and identify their potential for EOR applications.

In summary, carrier systems for surfactant delivery, whether supramolecular or nonsupramolecular, represent a promising class of technologies for the development of advanced EOR solutions. Their ability to form highly organized structures, respond to external stimuli, and incorporate additional functionalities makes these systems ideal for a wide variety of applications. This review addresses the development of smart surfactant carrier systems for EOR. Section 2 covers the main topics that can affect the effectiveness of surfactant as an additive in EOR and discusses mechanisms that can make surfactant injection unfeasible, covering also enviromental and economical aspects (Section 2.5). In Section 3, we review the most recent surfactant carriers, including inorganic nanoparticles (Section 3.1), carbon nanomaterials (Section 3.2), polymers (Section 3.3), and supramolecular carriers (Section 3.4), and discuss the most relevant aspects of each carrier-surfactant interaction.

## 2 Surfactants in EOR

Surfactants are amphiphilic organic compounds formed by a hydrocarbon chain (hydrophobic group – the tail) and a hydrophilic group (the head), being classified according to the nature of their head into anionic, cationic, nonionic, and zwitterionic. Nonionic surfactants have no charge; anionic surfactants have a negative charge on their polar head, while cationic surfactants have a positive one. On the other hand, zwitterionic surfactants have both a positive and negative charge on their polar head.^51,52^ Due to their chemical structure, surfactants can adsorb onto solid or liquid surfaces and at the solid/liquid and liquid/liquid interfaces, even at low concentrations. This significantly alters the physicochemical properties of the systems, such as reducing the IFT of water/oil interfaces and changing the wettability of rocks. These effects vary considerably above and below the critical micelle concentration (CMC). Above the CMC, surfactants in solution form larger aggregates known as micelles. In EOR applications, it is essential to inject surfactants above the CMC to achieve low IFT values, improve foam stability, and reduce adsorption on the rock surfaces.^53^

The hydrophilic–lipophilic balance (HLB) is a crucial factor characterizing surfactants, measuring their degree of hydrophilicity or lipophilicity. HLB values range from 0 to 20, with 0 indicating complete hydrophobicity and 20 representing total hydrophilicity. Surfactants with an HLB value below 9 are considered lipophilic, while those with values exceeding 11 are deemed hydrophilic. These HLB values play a vital role in determining the effectiveness and applicability of surfactants across various types of reservoirs. By utilizing HLB, one can anticipate the properties of surfactants, including their propensity to form water/oil or oil/water emulsions. Consequently, surfactants with higher HLB numbers exhibit enhanced solubility in water, favoring the formation of oil-in-water emulsions.

For surfactant application in EOR, the surfactant must have thermal stability at the reservoir temperature, reduce the oil/water IFT to 10^–3^ mN/m, have low retention in the reservoir rock (<1 mg/g of rock), have high tolerance to the reservoir salinity, commercial availability, and low environmental impact. Surfactants application in EOR has been deeply discussed in recent reviews.^53−55^ Therefore, this section will cover an introduction to the basic concepts to understand the properties and actions of the surfactant carriers.

### 2.1 Surfactants and Interfacial Tension

In chemical EOR projects involving surfactant application, the IFT is one of the main factors that need to be considered. IFT is the attraction force between molecules at the interface of two fluids that occurs due to the unbalanced attraction of molecules at the interface. The imprisonment of oil in the rocks’ pores is associated with capillary forces and the interfacial tension of the system. The greater the IFT between the fluids, the more significant the pressure that the injected fluid must overcome to displace the trapped fluid. To increase the oil recovery factor (ORF) and improve displacement efficiency, reducing capillary forces by decreasing the interfacial tension is necessary. Thus, IFT is one of the most critical parameters for EOR. Therefore, the main function of surfactants in EOR processes is to reduce the water/oil IFT, promoting an increase in the capillary number, a dimensionless parameter that relates a system’s viscous and capillary forces, and is inversely proportional to the IFT and the fluid/rock contact angle.^56^ The capillary number is given by Equation 1:
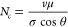
1where *N*_*c*_ is the capillary number, *v* is the injected fluid velocity calculated by Darcy’s law, μ is the viscosity of the injected fluid, σ is the water–oil IFT, and θ is the contact angle measured at the rock/oil/water interface.^56^ For the residual oil saturation to decrease, increasing the oil recovery factor, the capillary number must increase, reaching a critical value of 10^–2^.^3,7,57^

For oil-wet carbonate systems, capillary pressure is generally negative, preventing water from spontaneously absorbing into the porous medium because the oil is firmly trapped on the rock surface by capillarity. The use of surfactants in EOR reduces IFT and decreases the adhesive forces that retain the oil. This allows residual oil droplets to flow through pore throats and merge with the oil moving toward the low-pressure zone of the production well.^57^

The ability of surfactants to reduce IFT depends on ion concentration, requiring an optimal salinity for achieving ultralow IFT values.^57,58^ Akhlaghi et al. investigated the effect of monovalent and divalent ions, temperature, and pH on interfacial tension in brine/Triton-X/oil systems. They found that increasing the surfactant concentration, salinity, and pH in the aqueous medium reduced interfacial tension, while temperature had the opposite effect. Additionally, bivalent ions were more effective at reducing interfacial tension than monovalent ions. The reduction of IFT between oil and brine under specific temperature and salinity conditions leads to the formation of emulsions, thereby enhancing oil recovery after primary and secondary recovery stages.^59^ In this context, low-salinity water flooding is a cost-effective and environmentally friendly oil extraction method, supported by laboratory experiments and field trials. Additionally, combining low-salinity water injection with surfactants reduces IFT, thereby enhancing oil production rates.^60^

### 2.2 Surfactants and Reservoir Wettability

Wettability is another important factor that affects oil recovery rates. The wettability of a rock refers to the affinity or interaction between the fluids present in a rock formation and the rock itself.^61^ This phenomenon is crucial in oil exploration and production as it directly influences the movement and distribution of fluids, such as oil, water, and gas, in the subsurface.^62^

**Figure 1:**
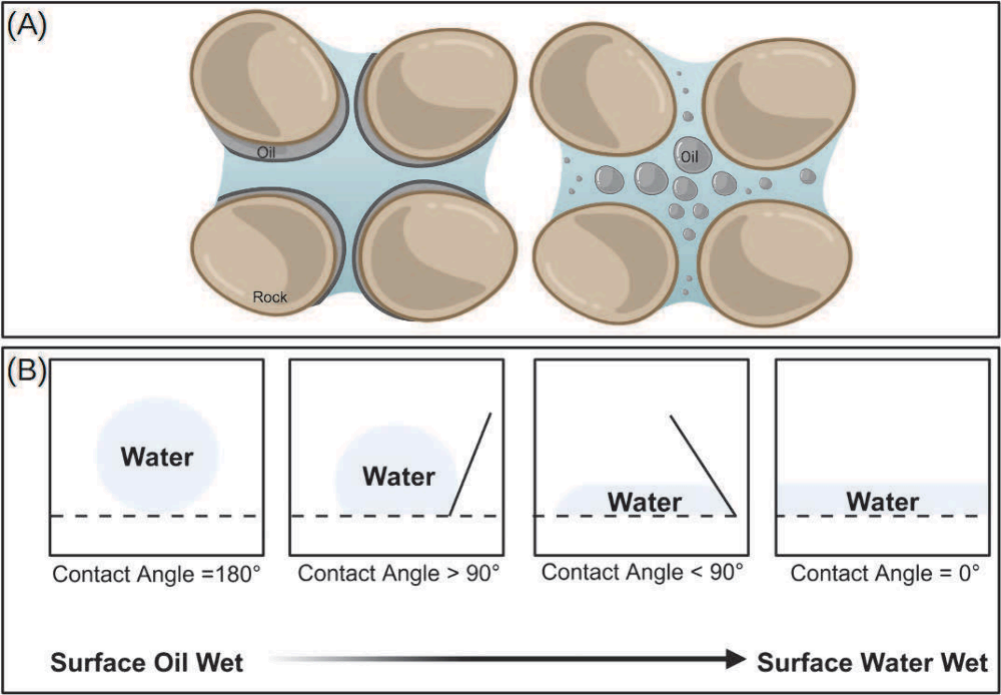
(A) Schematic representation of a reservoir with oil-wet rocks, and wettability alteration, allowing greater oil removal efficiency. (B) Wettability of a liquid on a surface as a function of the contact angle. Created using BioRender.com.

For an oil/water system, wettability is described in terms of the contact between the rock and the fluids, classified into three main types: oil-wet rocks, water-wet rocks, and rocks with intermediate wettability.^61^ As a surface becomes wettable to a liquid, it spreads and covers the solid, reducing the contact angle. When the rock is nonwettable to the fluid, the contact angle increases, and the formation of a spherical drop is favored as it represents lower energy configuration (Figure 1). As shown in Figure 1 the rock will be water-wettable when the contact angle is in the range of 0–75°, oil-wettable with an angle in the range of 115–180°, and additionally the rock may exhibit intermediate wettability in the range of 75–115°.^63^ It is worth noting that 50–60% of the world’s oil reserves are found in carbonate rocks, which have wettability ranging from neutral to oil-wet, due to prolonged exposure to oil.^64^

Surfactants can modify the reservoir’s wettability from oil-wet to mixed or even water-wettable. The wettability of an oil reservoir is complex and is related to the distribution of fluids in the pores, pore size, permeability, and mineralogical composition. The fluids’ behavior within the porous confirms that in a brine-oil-rock system, if water occupies the smallest pores and wets the surface of the larger pores, i.e., the rock is moistened with water. In this way, in areas of high oil saturation, the oil rests on a film of water spread on the surface of the larger pores. On the other hand, the rock will be preferentially oil-wettable in areas of high water saturation.^56^ Thus, the smaller pores will be soaked in oil, and the larger pores will be filled with water.^56^ Therefore, in the oil recovery process, the wettability of the reservoir rock influences the oil recovery factor in the way that water-wettable or mixed wettability reservoirs have a more significant recovery factor than oil-wettable reservoirs.^56^

**Figure 2:**
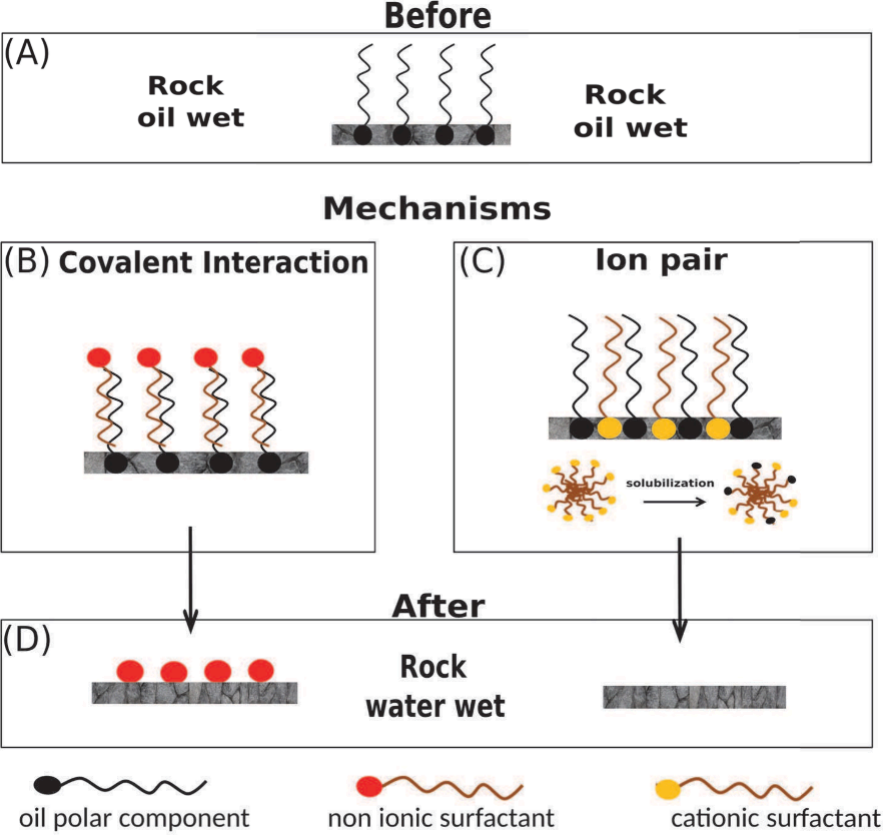
Rock wettability modification mechanisms. (A) Aging and oil-wetting of the rock, which is a natural process that occurs over time when oil displaces water from the rock’s surface. Surfactant action can be further divided into two submechanisms: (B) Nonionic surfactants adsorb onto the rock surface due to covalent interactions between the hydrophobic chain of the surfactant and the adsorbed oil. (C) Cationic surfactants interact electrostatically with polar compounds contained in the oil, causing desorption of the complex and subsequent solubilization. (D) As a result of cationic surfactant action, the rock surface can become water-wetted.

The mechanism of wettability alteration by surfactant injection depends on the type of surfactant used and can be explained by two main mechanisms: adsorption (coating) or cleaning of the rock surface (Figure 2). The coating mechanism involves the creation of a monolayer on the rock surface by anionic or nonionic surfactants. The adsorption of surfactants on solid surfaces can occur through the formation of aggregates, which can either form a monolayer (admicelle) or a bilayer (hemimicelle), depending on the surfactant concentration. The extent of surfactant adsorption can be quantified using Freundlich, Langmuir, and Temkin isotherms, as well as linear models. These isotherms can be determined by measuring surfactant concentration in static tests or dynamic core flooding tests.^65,66^ In this mechanism, the hydrophobic tails interact with the adsorbed oil while the polar head points to the liquid medium, resulting in the rock’s surface being covered with hydrophilic surfactant heads.^54^ This arrangement modifies the rock wettability to water-wettable conditions. However, it may not increase the reservoir displacement efficiency since oil desorption does not occur.

On the other hand, the cleaning mechanism is more related to cationic surfactants. In this mechanism, ionic pairs are formed between the surfactants’ cationic heads and the crude oil’s acid groups adsorbed on the rock surface.^54^ The ion pairs formed can strip the adsorbed oil layer from the rock surface, which is solubilized within the surfactant micelles, exposing the rock surface, which is originally water-wettable. For the success of the oil solubilization step within the surfactant micelles, the surfactant solution must be injected above its critical micelle concentration.^67^

The alteration of rock wettability by surfactant injection can be studied by atomic force microscopy (AFM) and contact angle measurements. Hou et al. investigated the ability of surfactants of different natures to change the wettability of sandstone rocks by analyzing a mica surface before and after exposure to the surfactant solution. The authors concluded that cationic surfactants, such as cetyltrimethylammonium bromide (CTAB), acted according to the cleaning mechanism, while anionic and nonionic surfactants coated the rock surface.^68^

It is worth noting that the success of wettability alteration process depends on the type of surfactant and the concentration of ions in the brine. Therefore, careful selection and optimization of surfactant concentration and injection strategy are crucial for achieving the desired wettability alteration and improving EOR efficiency.

In carbonate reservoirs, cationic surfactants are generally more effective in altering wettability than anionic surfactants due to the stronger ion-pair interactions.^55,69−73^ Furthermore, the desorption of oil fractions can increase their mobility in the porous medium. Studies have shown that modifying the polar head of cationic surfactants can further improve their efficiency in changing the wettability of carbonate rocks. Salehi et al. demonstrated that surfactants with a high charge density polar head were more effective in changing rock wettability to water-wet when ion pair formation was responsible for the wettability alteration. They propose that dimeric surfactants with two polar heads and hydrophobic tails can improve the wettability alteration process.^74−76^ Trimeric cationic surfactants, such as those containing three dodecyl chains and three quaternary ammonium head groups connected by divinyl groups, have also been evaluated as wettability modifying agents. Zhang et al. proposed two wettability change models for this surfactant that can alter wettability for either oil or water wettable, depending on its concentration.^77−80^ Their experimental findings demonstrate that wettability alteration mainly occurs due to the formation of ion pairs and the adsorption of surfactant molecules on the rock surface. It is important to note that the effectiveness of wettability modification heavily relies on the type of surfactant used.^57,81−85^

Using surfactants in EOR flooding can enhance oil displacement efficiency by reducing the IFT and changing wettability. Both of these effects can increase the system’s capillary number (Equation 1) and significantly increase the oil recovery factor. However, a recent study questioned the relative importance of these properties. In their article ”IFT or wettability alteration: What is more important for oil recovery in oil-wet formation?” Zhang et al. concluded that IFT reduction is more important for surfactants.^86^ Other authors have also critically reviewed the relative contributions of wettability change and IFT reduction in EOR and found that IFT reduction alone increases residual oil recovery in all wettability cases. Still, the effect of changing wettability depends on the initial wetting state.^87^ While surfactants have different mechanisms of action to reverse wettability, the efficacy of wettability alteration depends on the specific combination of surfactants and carrier agents used. In general, reducing interfacial tension is often more effective and pronounced than changing wettability.

### 2.3 Reservoir Mineralogy and the Surfactant Choice

The selection of the appropriate surfactant for EOR operations must consider the reservoir’s temperature and salinity conditions, the rock type, the order of magnitude of the IFT value, and the surfactant adsorption, among other factors.^54^

The characteristics of the reservoir will dictate the best surfactant to be employed. Although the majority of oil reserves are in carbonate reservoirs, most EOR projects have been developed in sandstone formations. A sandstone reservoir rock consists predominantly of silica with silicate and clay minerals like kaolinite and Illite. Sandstone reservoirs are more homogeneous than carbonate reservoirs, making them more suitable for chemical EOR. In contrast, carbonate reservoirs are highly heterogeneous, fractured, and surrounded by a low-permeability matrix. Carbonate rocks are composed of minerals such as calcite (*CaCO*_3_), dolomite , anhydrite (*CaSO*_4_), gypsum (*CaSO*_4_ · *H*_2_*O*), and magnesite (*MgCO*_3_), and exhibit mixed or oil-wet wettability.^88^

Despite containing large oil reserves, the application of EOR techniques in carbonate reservoirs is limited and less effective due to several technical challenges. These include high clay content leading to significant surfactant adsorption and the precipitation of calcium carbonate and calcium hydroxide resulting from reactions between injected surfactants and divalent ions (*Ca*^2+^ and *Mg*^2+^). The surface charge of the rock at the solid–liquid interface depends mainly on pH and ionic strength. Silica and calcite are typically used as representative surfaces for sandstone and carbonate formations, respectively.^89,90^

At the isoelectric point (IEP), the surface carries no net electrical charge. For silica, this occurs at pH 2, and for calcite, at pH 9. Below the IEP, the surface is positively charged, and above the IEP, it is negatively charged. Therefore, at pH close to 7, carbonate rock has a positive charge, while sandstone has a negative charge. This surface charge directly influences the surfactant adsorption processes, as discussed further in this review, and will indicate which surfactant is best used in injection. Thus, considering ways to minimize surfactant loss through adsorption, the results show that higher oil production rates should be obtained when surfactant injection prioritizes electrostatic repulsion between the surfactant and the rock surface.^91^ Therefore, it is required that injection into carbonate reservoirs be carried out with cationic or nonionic surfactants, possessing high HLB values so that the formed emulsions remain stable in saline environments, do not break due to electrostatic attraction, and prevent surfactant adsorption onto the rock. The opposite applies to surfactant injection into sandstone formations.^92,93^

Anionic surfactants, including sulfonates, sulfates, and carboxylates, are commonly used in EOR in sandstone formations. Sulfonate surfactants are resistant to high temperatures but not tolerant to high salinity, limiting their use in low-salinity environments.^81^ Surfactants containing sulfate groups have greater tolerance to divalent cations but decompose at temperatures above 60^*o*^*C*.^94^ Guerbet alkoxy sulfate (GAS) surfactants can reduce IFT to ultralow values at high temperatures with different types of crude oil, while alkoxy carboxylate surfactants are stable at high temperatures and generate ultralow IFT with low viscosity emulsions. Both types of surfactants have demonstrated excellent performance in sandstone and carbonate reservoirs.^53,81,95,96^ In contrast, cationic surfactants are normally used in carbonate reservoirs, with the derived from quaternary ammonium salts, gemini bis (quaternary ammonium bromide) surfactants, cetylpyridinium chloride, and dodecyltrimethylammonium chloride the most commum ones.^53,93,97^

Zwitterionic surfactants have attractive advantages under high-temperature and high-salinity conditions and also possess very low CMC values. However, they are more expensive compared to other surfactants. The zwitterionic surfactants that have been evaluated for EOR applications include the following: carboxyl betaine surfactants, hydroxyl sulfonate betaine, didodecylmethylcarboxyl betaine, alkyl dimethylpropane sultaine, lauramidopropyl betaine, and cocoamidopropyl hydroxysulfobetaine.^56,92,98^

Nonionic surfactants are commonly used as cosurfactants in EOR due to their high chemical stability and salinity tolerance. While they do not decrease interfacial tension to the same extent as ionic surfactants, they are still effective in EOR applications. Ethoxylated alcohols, alkyl polyglycosides, ethoxylated nonylphenol, polyethylene glycol derivatives, and copolymers like Triton-X are some of the nonionic surfactants used in EOR.^99,100^

In addition to the previously mentioned classes, there are other special categories of surfactants, including viscoelastic surfactants, natural surfactants, and polymeric surfactants. Polymeric surfactants exhibit dual characteristics, functioning as both surfactants and polymers. As a result, they can be classified either as surfactants or polymers, depending on the author’s focus. Therefore, the details about polymeric surfactants will be discussed in Section 3.3.

Viscoelastic surfactants, similar to polymeric surfactants, combine the properties of surfactants and polymers. These surfactants form elongated micelles with a supramolecular structure, resulting in significantly high viscosity in aqueous solutions.^101,102^ Viscoelastic surfactants can be ionic or zwitterionic, and their viscoelastic properties are determined by their molecular structure. Therefore, they act mainly enhancing the sweep efficiency and oil displacement.^101,103−105^

In summary, surfactant injection reduces interfacial tension, changes wettability, and increases the capillary number, thereby increasing the recovery factor of the oil retained in the reservoir’s pores. Table 1 presents the relationship between the surfactant, their mechanism of action, and advantages for EOR. However, the injection of free surfactant during the EOR process can become economically unfeasible due to their adsorption on the rock surface, which leads to several losses.^106,107^ The next section will discuss the main causes of surfactant loss during EOR flooding and strategies to minimize it.

**Table 1:** Relationship of the Type of Surfactant with the Advantages and the Mechanism of Action in the EOR^a^

Surfactant Type	Type of Reservoirs	Advantages	Mechanism
Cationic	Limestone	Stable solutions in brine; can be blended with nonionic surfactants synergistically.	IFT Reduction alteration of wettability
Anionic	Sandstone	Stable at high temperatures	IFT reduction
Nonionic	Carbonate, siliceous and carbonate shale cores	Effective in floods containing high salinity systems and severe pressure and temperature conditions	Small IFT reduction. Formation of stable *CO*_2_ foams in brine
Zwitterionic	Carbonate	Low CMC. High thermal stability and high salinity tolerance. Adsorption controlled by alkaline injection.	IFT Reduction alteration of wettability

a Adapted from ref (107). Licensed under a Creative Commons Attribution (CC BY) license.

### 2.4 Mechanisms of Surfactant Loss and Minimization Strategies

The adsorption process is the primary mechanism for surfactant loss during EOR flooding, with surfactants being retained on the injection well surface before reaching the reservoir.^34^Figure 2 illustrates the generic process of surfactant adsorption on a rock surface, which can be governed by electrostatic attraction, van der Waals interaction, and hydrogen bonding,^108−110^ and can lead to formation of surfactant aggregates such as hemimicelles, admicelles and/or micelles.^111,112^ Other interactions, such as hydrophobic interactions, dispersion forces, and π stacking, can also contribute to surfactant adsorption, resulting from a single type of interaction or a combination of two or more.^54,113^

The type of rock and surfactant used in EOR operations influence the adsorption process. As discussed, in pH close to 7, carbonate rocks have a positive surface charge, while sandstone has a negative charge.^114−116^ Therefore, cationic surfactants are generally used in carbonate reservoirs and anionic surfactants in sandstone reservoirs to minimize losses due to electrostatic interactions at the interface.^114^ However, adsorption cannot be neglected when rocks contain silica or clay impurities.^114^ For example, in natural carbonate clay, impurities containing silica and aluminum oxide introduce negative surface sites that attract cationic surfactants, resulting in greater surfactant adsorption and decreasing the wettability alteration by the surfactant action.^93,117,118^

Changing the pH of the medium can alter the surfactant’s adsorption mechanism by changing the rock’s surface charge. For instance, at pH close to 7, clays have a positive charge on the edges and a negative charge on the face, resulting in the adsorption of both cationic and anionic surfactants.^113^ Adding alkali (such as *Na*_2_*CO*_3_ or NaOH) to the medium can significantly reduce adsorption. A pH above 10 can decrease the positive charge at clay edges, reducing the retention of anionic surfactants by electrostatic repulsion.^119^ Studies have also shown that adding other alkalis, such as sodium silicate, sodium tripolyphosphate, and sodium tetraborate, can reduce surfactant adsorption.^92,119,120^

The retention of surfactants in carbonate reservoirs is generally higher than in sandstone reservoirs.^121−123^ This is due to the high density of positive charges on the surface, the presence of divalent ions, and the high degree of heterogeneity of the carbonate rock, which leads to phase trapping and more significant surfactant retention.^119,123^

Severe conditions of temperature, salinity, and the presence of divalent cations favor the adsorption of surfactants, particularly anionic ones.^114,124−126^ The interaction between anionic surfactants and salt ions can result in precipitation. According to the DLVO theory, at every charged interface, an electrical double layer is formed.^127,128^ The thickness of this layer is inversely proportional to the ionic strength of the medium, so the denser this layer will be, the greater the salinity. Thus, in high salinity mediums, there is a decrease in the distance between the surfactant and the surface, causing the attractive forces to predominate over the repulsive ones, leading to increased surfactant adsorption.^127,128^ However, extensions of DLVO theory should be considered to not underestimate (overestimate) the disjoining pressure at high (low) surfactant concentrations.^128^

Studies have shown that the adsorption of the anionic surfactant diphenyl ether disulfonate/alpholfinsulfonate (DPES/AOS) in sandstone is small under low salinity conditions and increases with the addition of divalent ions due to the effect of ionic strength.^129^ Using complexing or chelating agents, such as EDTA and polyphosphates, also minimizes surfactant loss. The presence of a chelating agent will cause the divalent cations to be complexed, decreasing the ionic strength and increasing the electric double-layer thickness. Thus, the surfactant loss by adsorption and precipitation is reduced.^53,120,130−132^

Mechanisms responsible for surfactant retention include surfactant precipitation,^94,133,134^ phase capture,^34,94,135^ surfactant diffusion in the pores,^34,95^ and partition of the surfactant in the oil phase.^34,136^ Injection of surfactants incompatible with severe temperature and salinity conditions can lead to precipitation and phase entrapment, so special surfactants are required. Phase entrapment may be due to the formation of micro or macroemulsions with low mobility, resulting in flow problems in highly heterogeneous reservoirs such as carbonate ones.^54,137^ This way, to optimize the oil recovery process, it is necessary to minimize the surfactant loss. Strategies to reduce surfactant loss during EOR flooding include changing pH using ammonia,^138^ the use of cosolvents,^139,140^ sacrificial nanoparticles,^141,142^ surfactant carriers, and ionic liquids.^143,144^ Nanoparticles can act as a sacrifice adsorption agent, while ammonia can increase the pH and reduce the loss of anionic surfactant in sandstone rocks without causing precipitation of *Ca*^2+^ ions. Co-solvents can prevent surfactant loss by phase entrapment, forming low-viscosity microemulsions.^139,140^

**Figure 3:**
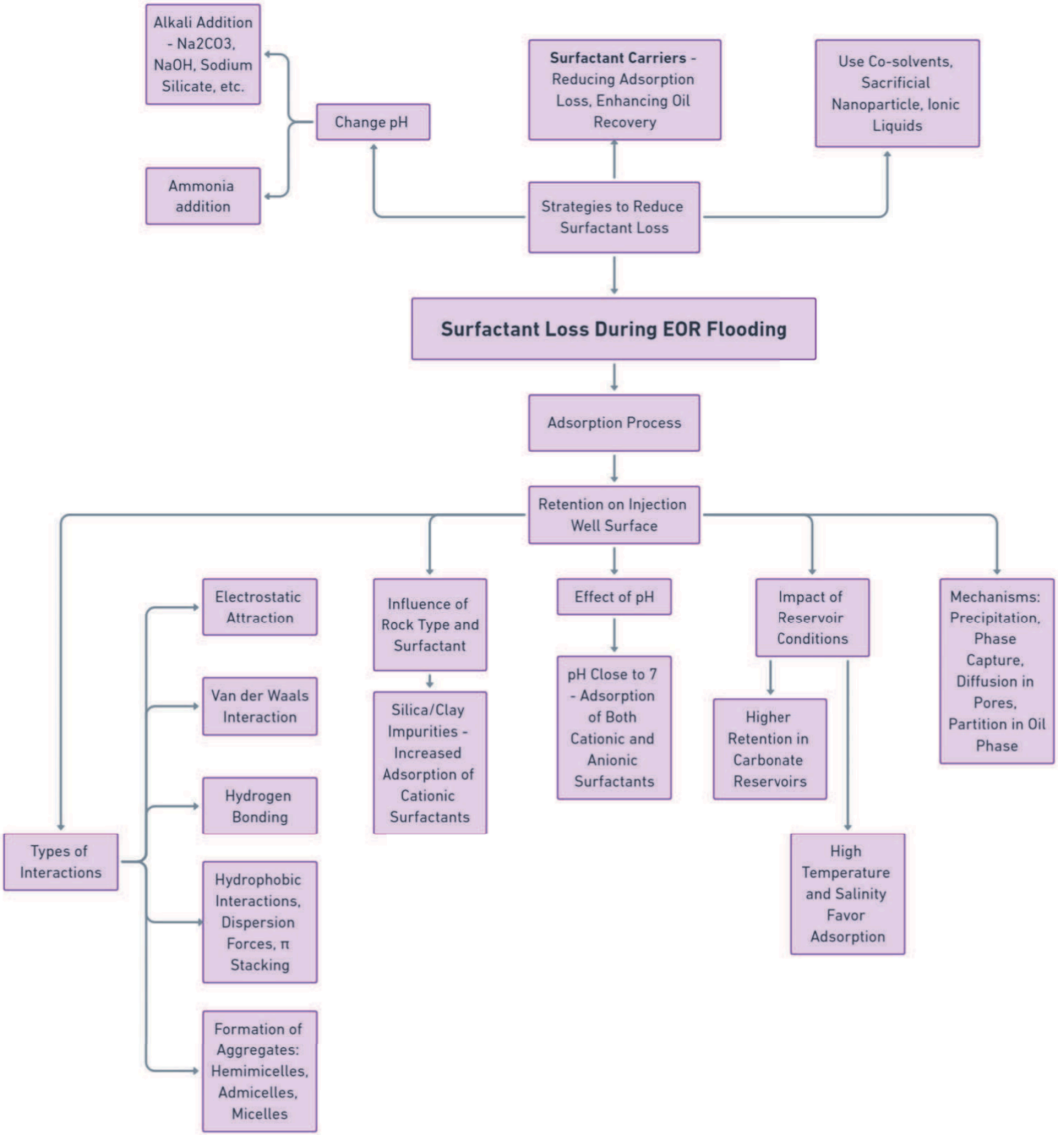
Surfactant loss during EOR. Flowchart illustrating the adsorption process of surfactants in EOR, and the optimization of the EOR process to minimize surfactant loss.

The flowchart in Figure 3 illustrates the adsorption process of surfactants in EOR, and the optimization of the EOR process to minimize surfactant loss. Surfactant carriers are an innovation in the EOR area with high potential for application. These systems can increase the oil recovery factor by reducing surfactant loss through adsorption. The Section 3 will discuss the main surfactant carrier systems, their carrier mechanism, and how they can act synergistically as EOR agents.

### 2.5 Environmental Effects and Economical Analysis

Surfactant toxicity in aquatic environments has been a subject of considerable scientific inquiry, with studies exploring the multifaceted impacts of these compounds on various organisms and ecosystems. Several reviews provide comprehensive insights into the diverse effects of surfactants on aquatic life.^145−147^

Factors such as biodegradability and persistence influence their impact on ecosystems. Certain surfactants can persist in the environment, raising questions about their long-term effects on terrestrial and aquatic ecosystems. Cationic surfactants are widely used in EOR formulation despite being more toxic than anionic or nonionic ones. However, some anionic surfactants, particularly linear alkylbenzene sulfonates (LAS), exhibit increased persistence in aquatic environments, elevating the potential risks associated with their presence.^146^

One of the central concerns highlighted in ecotoxicological studies is the concentration-dependent nature of surfactant toxicity. While these compounds enhance the effectiveness of various products, even chronic exposure to low concentrations can lead to adverse effects on aquatic ecosystems. Generally, surfactant toxicity is related to the presence of free monomers in solution. Therefore, a EOR formulation with surfactant concentrations higher than the CMC can decrease the toxic effects.^148^ In this sense, the use of surfactant carriers can decrease their toxicity by decreasing the free monomers concentration. However, in each case the biocompatibility of the carriers should also be addressed, as will be discussed in the next sections.

In aquatic environments, surfactants have been shown to have detrimental effects on organisms such as fish, invertebrates, and algae. Disruptions in physiological processes, altered behavior, and reproductive issues are among the documented consequences, emphasizing the need for a nuanced understanding of their impact on aquatic ecosystems. Fish, being particularly vulnerable, experience disruptions in gill function, respiratory distress, altered behavior, and compromised reproductive and developmental processes.^145−147,149−151^ Furthermore, the impact of surfactants extends to invertebrates, with crustaceans and insects demonstrating sensitivity to these compounds. The consequences include physiological disruptions that can contribute to population declines, thereby affecting the overall biodiversity and ecological balance within aquatic ecosystems.^145−147,152^

When surfactants are introduced into the soil, they can alter the physical and chemical properties of the soil matrix. For instance, surfactants can change soil structure by affecting soil aggregation and porosity. These changes can influence water infiltration rates and soil aeration, which are critical for maintaining soil fertility and supporting plant growth.^146,153^ Additionally, surfactants can affect soil microbial communities, which play a crucial role in nutrient cycling and organic matter decomposition. Some studies have shown that surfactants can inhibit microbial activity and reduce microbial diversity, leading to a decline in soil health and productivity.^153,154^ The alteration of microbial communities can also impact the degradation of organic pollutants in the soil, potentially leading to the accumulation of harmful substances.

The emergence of antibacterial surfactants has introduced another dimension to the discussion. Concerns about antibiotic resistance have prompted a cautious approach to their use in consumer products. Striking a balance between the benefits of antibacterial surfactants and the potential risks of resistance is crucial in navigating this aspect of surfactant toxicity.

**Figure 4:**
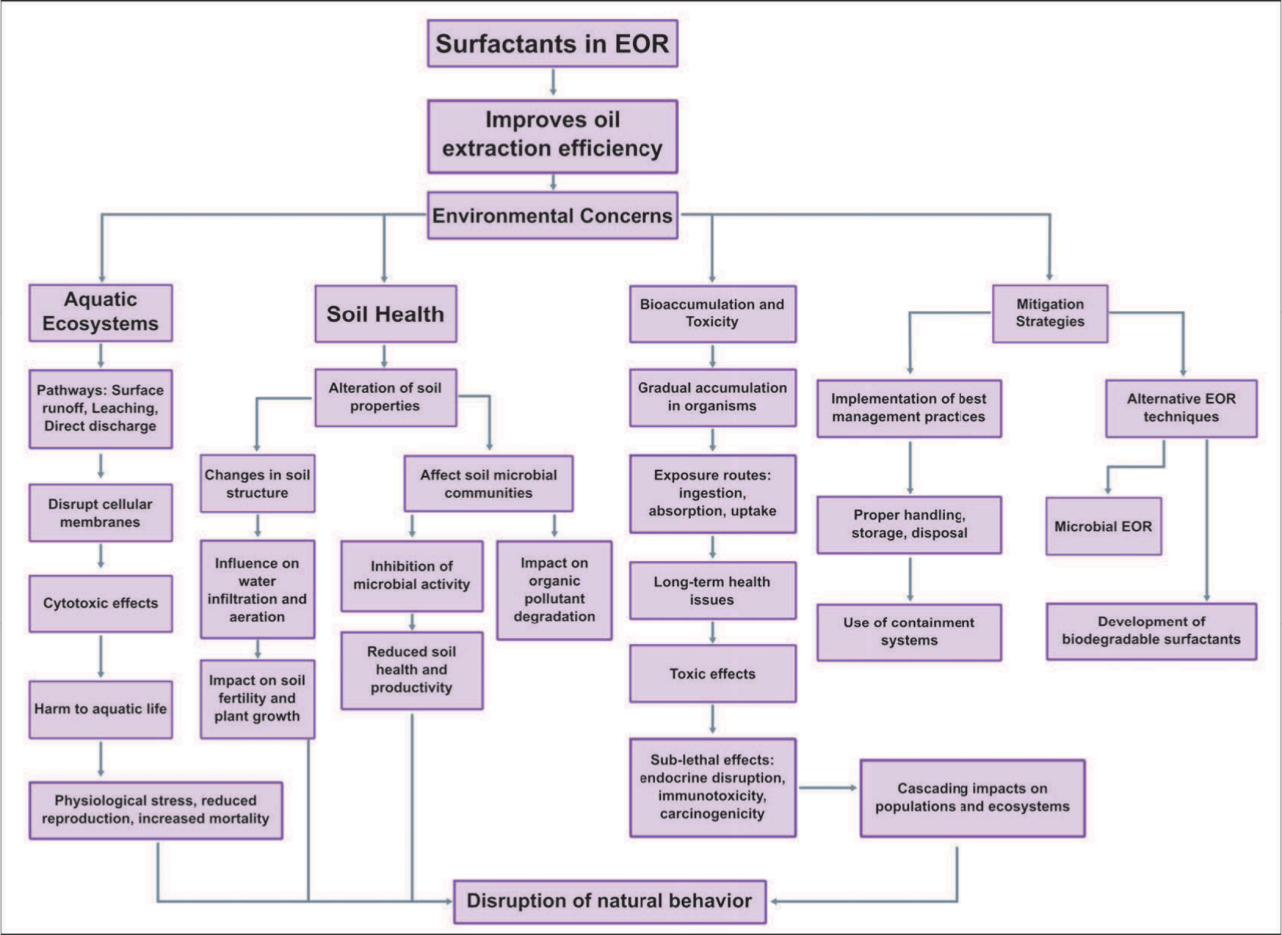
Environmental concerns of surfactants in EOR. This flowchart illustrates the environmental effects of surfactants used in enhanced oil recovery (EOR). It begins with the use of surfactants in EOR to improve oil extraction efficiency, leading to significant environmental concerns. These concerns are categorized into three main areas: the impact on aquatic ecosystems, soil health, and the potential for bioaccumulation and toxicity. Each category outlines the pathways, effects, and consequences of surfactant use, highlighting the disruption of cellular membranes in aquatic life, alteration of soil properties, and accumulation of surfactants in organisms. The flowchart also presents mitigation strategies, including the development of biodegradable surfactants, best management practices, and alternative EOR techniques such as microbial EOR, to reduce the environmental footprint of surfactants.

Another significant environmental concern is the potential for bioaccumulation of surfactants. Bioaccumulation refers to the gradual accumulation of substances, such as surfactants, in the tissues of living organisms. This process can occur through various exposure routes, including ingestion, dermal absorption, and respiratory uptake. Once accumulated, surfactants can exert toxic effects on organisms, potentially leading to long-term health issues.^155^ The toxicity of surfactants can vary depending on their chemical structure and concentration. For example, nonionic surfactants, while generally less toxic than anionic surfactants, can still pose risks to organisms at high concentrations. Chronic exposure to surfactants can lead to sublethal effects, such as endocrine disruption, immunotoxicity, and carcinogenicity. These effects can have cascading impacts on populations and ecosystems, highlighting the need for comprehensive risk assessments and regulatory measures (Figure 4).

To address these concerns related to surfactant toxicity, regulatory frameworks and guidelines have been implemented globally. Regulatory agencies set limits on the use of specific surfactants in consumer products, aiming to safeguard both human health and the environment. Compliance with these regulations is vital to mitigate potential risks associated with surfactant exposure. However, despite recent progress in the development of new surfactants for EOR, more comprehensive toxicological studies and specific regulatory frameworks should be addressed.

While regulatory measures play a pivotal role in mitigating surfactant-related environmental risks, the pursuit of sustainable practices involves not only monitoring and restriction but also innovation. The development of alternative surfactants, as explored in current research, aims to strike a balance between the benefits of surfactant use and the imperative to safeguard aquatic ecosystems. Green surfactants, derived from renewable resources,^156−158^ and biosurfactants^159−161^ represent a promising avenue for minimizing environmental impact and fostering ecologically responsible practices in EOR. However, synthetic surfactants still are more cost-effectiveness, have extended shelf life, widespread availability, and superior performance in comparison to biodegradable surfactants. Although certain natural and biodegradable surfactants possess favorable physicochemical properties and hold promise as ecologically sustainable alternatives for the future, their application on a large scale is impeded by lower efficiency and elevated costs. Consequently, these factors diminish their attractiveness for widespread adoption within the industrial sector.

In conclusion, the discourse on surfactant toxicity underscores the need for a holistic understanding of the various dimensions involved. From the chemical classification of surfactants to their ecological impacts on fish, invertebrates, and overall ecosystem dynamics, research continues to shape our awareness of the challenges and opportunities in managing surfactant-related environmental risks. The integration of regulatory measures, informed by scientific insights, and the exploration of sustainable alternatives collectively contribute to a comprehensive approach aimed at preserving the health and integrity of aquatic ecosystems.

#### 2.5.1 Natural Surfactants

Environmental concerns and the toxic nature of chemical surfactants used in EOR have become major areas of interest for researchers. Consequently, studies focused on developing natural surfactants as alternatives to chemical surfactants are increasing.^90,162^ According to Holmberg, a natural surfactant is any surfactant produced directly from a plant or animal source.^163^ Plant extracts from leaves, roots, and bark contain natural surfactants called saponins, which can reduce the IFT, oil/rock surface contact angle, and aid in foam formation and emulsification. However, natural surfactants may have limitations such as sensitivity to high temperatures, salinity, and pH variations, which can affect their performance and foam stability. Like chemical surfactants, natural surfactants are classified based on the charge of their hydrophilic head.

A recent literature review conducted by Hama et al. highlights the application of natural surfactants in EOR.^90^ This review presents various types of natural surfactants, state-of-the-art techniques, and future perspectives. Notable examples include cationic surfactants extracted from plants such as Seidlitz rosmarinus, mulberry, and henna leaves. Anionic surfactants are obtained from plants like castor oil, palm oil, coconut oil, cashew nut shell liquid, and mahua oil. Sarkar et al. investigated a newly derived anionic natural surfactant from peanut oil and found that the IFT decreased from 15.5 mN/m to 6.8 × 10^–2^ mN/m.^164^ Additionally, the oil/surface contact angle decreased from 144° to 58° on carbonate rock and from 138° to 44° on sandstone rock, with oil recovery increasing by 19.3% and 15.64% of original oil in place (OOIP) from the sandstone and carbonate core plugs, respectively.^164^

Nonionic surfactants from plants include *Matricaria chamomilla*, *Zizyphus spina-christi*, *Glycyrrhiza glabra*, and the soapwort plant where demonstrated by Eslahati et al. In their research they evaluated a nonionic surfactant obtained from the alfalfa plant in fractured, moderately oil-wet carbonate reservoirs. They observed a 63.39% reduction in IFT, a 49.91% alteration in wettability, and a 62% increase in OOIP oil recovery.^165^ A study led by Abbas Khaksar Manshad et al. examined the efficacy of two environmentally friendly surfactants, Hop and Dill, in reducing the IFT.^166^ The findings indicated a significant reduction in initial IFT values, decreasing from 28 to 2.443 mN/m for Hop and 5.614 mN/m for Dill. Furthermore, coinjection with low-salinity water optimized their effectiveness, resulting in oil recovery rates of 8.56% for Hop and 10.11% for Dill.

Zwitterionic surfactants, which are thermally stable and salt-tolerant, can be produced from lignin, castor oil, cashew nut shell liquid, and residual cooking oil. In 2018, Xu et al. developed a novel biobased zwitterionic surfactant from transgenic soybean oil using a simple two-step reaction involving amidation and quaternization. This surfactant showed a CMC as low as 33.34 mg/L at a IFT of 28.50 mN/m, demonstrating high interfacial activity in aqueous solutions containing *Na*_2_*CO*_3_.^167^

The potential of different natural surfactants to increase oil recovery in EOR applications has been evaluated. Extracts from Tanacetum and Tarragon plants showed recovery rates of 13.20% and 11.70%, respectively. Additionally, passiflora plant extract was used as a natural surfactant in EOR applications, facilitating an additional 7.5% of oil extraction.^168^

Despite the environmental benefits, natural surfactants must overcome technological challenges such as cost-effectiveness in extraction and production, large-scale availability to meet market demand, and other factors to ensure their commercial viability. Continued scientific investigation and technological advancements are necessary to address these challenges.^90^

## 3 EOR Surfactant Carriers

The primary objective of a surfactant delivery system is to transport the surfactant to the oil–water interface through the porous medium while minimizing adsorption losses on the rock surface.^169−171^ The surfactant carriers should possess specific properties, including a strong interaction between the carrier and surfactant that is stronger than the surfactant’s interaction with the rock surface. The carrier-surfactant complex should be small enough to penetrate through the reservoir pores without affecting the rocks’ porosity. Moreover, the carrier must release the surfactant only at the target site, and for this, the surfactant-oil interaction should be stronger than the surfactant-carrier interaction.

To decrease surfactant loss during the EOR flooding process, systems capable of carrying surfactants based on organic or inorganic materials, lipid matrices, polymers, and supramolecular systems have been proposed as simple and effective strategies.^4,38,39,61,172−176^ Furthermore, several studies have shown that surfactant carriers can result in controlled release of surfactant and act synergistically with the surfactant, reducing interfacial tension and making the rock even more water-wettable.^37,177,178^

Nanomaterials are promising surfactant carriers for reducing surfactant losses. Although inorganic nanoparticles (NPs) have been extensively studied in advanced oil recovery, their use as surfactant carriers is limited in the literature. Polymeric NPs, on the other hand, have high potential for EOR applications but are still poorly explored.^34,38,54,106^ In the following sections, we will review the application of polymers and inorganic NPs, carbon nanomaterials, and supramolecular systems as surfactant carriers for EOR.

### 3.1 Inorganic Nanoparticles

Nanoparticles possess unique properties that have made them attractive for use in several technology branches. Regarding their synthesis methodologies, the chemical synthesis of inorganic nanoparticles through colloidal processes stands out as one of the most prevalent techniques due to its cost-effectiveness, ease of implementation, and scalability. A plethora of methods exists for nanoparticle synthesis, including chemical reduction, electrochemical reduction, thermal decomposition, photochemical decomposition, hydrothermal synthesis, sol–gel processes, microemulsion techniques, coprecipitation, and others.^179^ Precise control over the composition, size, and shape of nanoparticles necessitates meticulous management of both nucleation and growth stages, achieved through manipulation of the kinetics and thermodynamics governing each reaction step.^180^

Utilizing surfactants as stabilizing agents with a high affinity for the nanoparticle surface enables fine-tuned control over the growth stage, thereby influencing nanoparticle morphology. Moreover, this results in the formation of surfactant-nanoparticle carrier complexes during the synthesis process. This synthesis approach has garnered significant attention in research circles, prompting the emergence of numerous comprehensive reviews elucidating the principles underlying surfactant-nanoparticle synthesis and chemical modification.^181−183^

In EOR, nanoparticles can improve the oil recovery factor through multiple mechanisms such as wettability inversion via adsorption or disjoining pressure created at the rock/oil/water interface, pore channel plugging, IFT reduction at the oil/water interface, and inhibition of asphaltene precipitation.^33,39,46,175,184−188^

**Figure 5:**
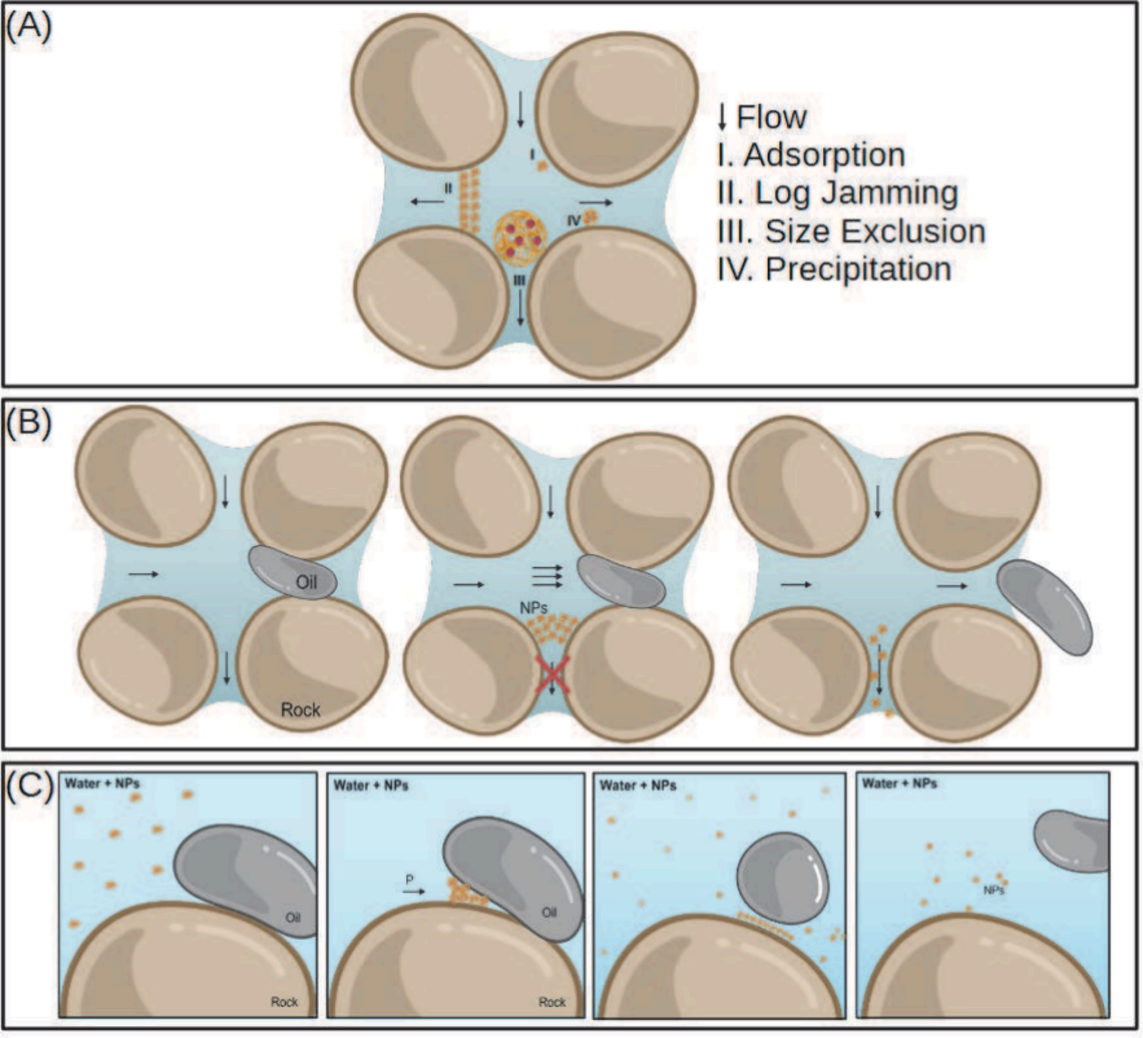
(A) Mechanisms of nanoparticle retention in porous media. I) Adsorption. II) Log-jamming (congestion). III) Size exclusion. IV) Precipitation. (B) Temporary NP congestion in pores showingoOil in path of lower permeability, the pore throat blockage and capillary flow redirection, and the oil extrusion and increased sweep efficiency. (C) Mechanism of wettability alteration of rocks by nanoparticles and release of adsorbed oil from rock through the disjoining pressure mechanism. Created with BioRender.com.

Due to the reservoir porosity, nanoparticles can be retained in reservoirs in four main ways: adsorption, log-jamming (congestion), size exclusion, or precipitation (see Figure 5).^189^ These mechanisms act on pore obstruction and rock wettability, directly affecting permeability and oil recovery. In this context, adsorption (Figure 5A I) is the process by which nanoparticles bind to the rock surface. This can occur through physical or chemical interactions.^190^ This mechanism is directly related to the wettability inversion of the rock and the occurrence of disjoining pressure, as will be further discussed.

“Log jamming” (Figure 5A II) occurs when there is an excessive accumulation of NPs in the pores, leading to flow obstruction. This can be caused by nanoparticle agglomeration or differences in velocity while passing through pore throats.^190^ Size exclusion (Figure 5A III) occurs when nanoparticles are too large to pass through smaller pores, being blocked due to their size relative to pore dimensions.^190^ This phenomenon underscores the importance of knowing the sizes of NPs, permeability, and porosity of the rock. These two mechanisms affect rock permeability as well as wellbore pressure.

When a pore is obstructed, fluid flow is altered, and consequently, the pressure in adjacent pores is increased. This pressure forces oil removal in neighboring regions, increasing the recovery factor, as showed in Figure 5B. As soon as the oil is removed from around the obstruction, the pressure decreases, and the pore blocked by congested NPs is gradually unblocked.^190^ Z. Hu et al. synthesized *TiO*_2_ NPs with a size distribution of 100–400 nm and noted that among other phenomena, log-jamming was responsible for increased oil recovery in core flooding tests.^191^ Additionally, when the authors evaluated NP transport in the porous medium (with 6% of pores smaller than 220 nm), only smaller and larger particles were recovered, while intermediate particles were retained, resulting in a bimodal distribution of recovered particles. This result indicates the importance of size and size distribution.

Nanoparticle precipitation in a reservoir rock (Figure 5A IV) is the process in which these particles deposit on the porous surfaces of the rock due to factors such as gravity or nanoparticle agglomeration.^190^ This retention form is also related to phenomena derived from adsorption.

As we discussed in Section 2 the wettability alteration is crucial in oil exploration an production, and is promoted when the oil-rock interaction is reduced and the oil–water interaction is promoted. This can be achieved by surfactants, as discussed, and by nanoparticles. The mechanism of action of nanoparticles on wettability is related to their ability to coat the rock surface in a thin layer, thereby displacing the oil and preventing new droplets from adsorbing to the surface. For this coating to occur, a “cleaning” of the rock surface takes place by the NPs due to the induction of disjoining pressure,^192^ as demonstrated in Figure 5C. Nanoparticles form a self-organized film at the oil/rock/water interface. This film grows and takes the shape of a prism, like a wedge (Figure 5C).^187^ Thus, pressure is exerted to separate the formation fluids from the rock, removing the layer of oil adhered to the reservoir surface and consequently increasing the percentage of oil recovered.^193^ This mechanism may also be responsible for altering the wettability of the rock by promoting the release of oil adsorbed to the surface.^190^ The smaller the size and the greater the quantity of particles in the wedge, the greater the resultant force and disjoining pressure.^190^

In general, particle size, cosurfactants, pH value, and ionic strength have been shown to influence wettability change.^131,194,195^ Karpor et al. observed changes in wettability on carbonate surface when zirconium oxide nanofluid was used. Their studies showed that wettability modification occurred due to the deposition of *ZrO*_2_ on the rock surface, governed by the nanomaterials partition coefficient in water and oil phases.^196^

Zhang et al. concluded that the disjoining pressure is the main mechanism underlying successful oil displacement in sandstone aged with crude oil when using silica nanoparticles. The pressure magnitude is related to the wedge film thickness, nanoparticle size, and structure. The contribution of each parameter to the disjoining pressure can be theoretically evaluated by solving the Ornstein–Zernike statistical mechanics equations.^187,197^ However, there is a lack of experimental studies showing the relationship between particle shape and disjoining pressure, wettability inversion, and oil recovery factor.

Particle morphology, surface functionalization and coating, and resistance to adverse conditions are factors that affect nanofluid quality as an EOR agent. Nanoparticles can achieve free movement in suitable oil reservoirs of different permeabilities with their small size and various geometries and dimensions without blocking the pore throats.^46,197^

Iron oxide nanoparticles (NPs) have received much attention due to their unique characteristics such as magnetism, low toxicity, and simple synthesis. These properties make them highly desirable to a wide range of researchers for applications such as magnetic fluids, data storage, catalysis, and bioapplications like drug delivery systems.^198,199^ Yahya et al. have demonstrated that cobalt-doped ferrite nanoparticles can act as hyperthermic agents, offering a promising technique for heavy oil recovery. The magnetic particle self-heats under high-frequency magnetic fields, changing the local water–oil viscosity and interfacial tension, resulting in increased oil recovery.^200,201^ Furthermore, magnetic nanoparticles have additional advantages such as targeted adsorption, remote sensing, directional transport, and local heating.^202^

However, the low stability of individual particles in a high saline environment leads to particle aggregation, which may cause pore obstruction and possible formation damage.^131,203,204^ For instance, Izadi et al. observed a pressure increase during coreflooding experiments with *Fe*_3_*O*_4_ due to porous blockage by nanoparticle aggregation and precipitation.^205^ To address this issue, the formulation of nanofluids containing inorganic NPs and surfactants has emerged as an effective strategy to improve particle stability under high salinity, temperature, and pressure conditions and to minimize surfactant loss through adsorption on the rock’s surface. In general, surfactant-coated nanoparticles outperform the use of surfactants alone, as they are responsible for specific tasks such as surfactant-controlled delivery at the oil–water interface, changing the IFT and the rock wettability, and optimizing the oil recovery process.^120,194,206−208^

A recent review by Dexin Liu et al. described the behavior of nanoparticle-surfactant-based nanofluids. This study demonstrates that nanofluids composed of nanoparticles and surfactants can enhance oil recovery on two fronts: by displacing oil through both surfactant and nanofluid action simultaneously, offering significant application potential.^50^

The formulation of high-performance nanoparticle-surfactant fluids involves three key steps, including the combination method and material selection principles. First, selecting nanoparticles with superior stability and efficiency in oil displacement, favoring those with smaller sizes and higher surface charges, to minimize the log jamming process and maximize the disjoining pressure. Second, choosing the type of surfactant with the highest CMC, the appropriate binding mode based on particle properties, and the reservoir mineralogy, as discussed in Section 2. Third, determining and controlling the concentrations of particles and surfactants to prevent double-layer adsorption during oil displacement.

**Figure 6:**
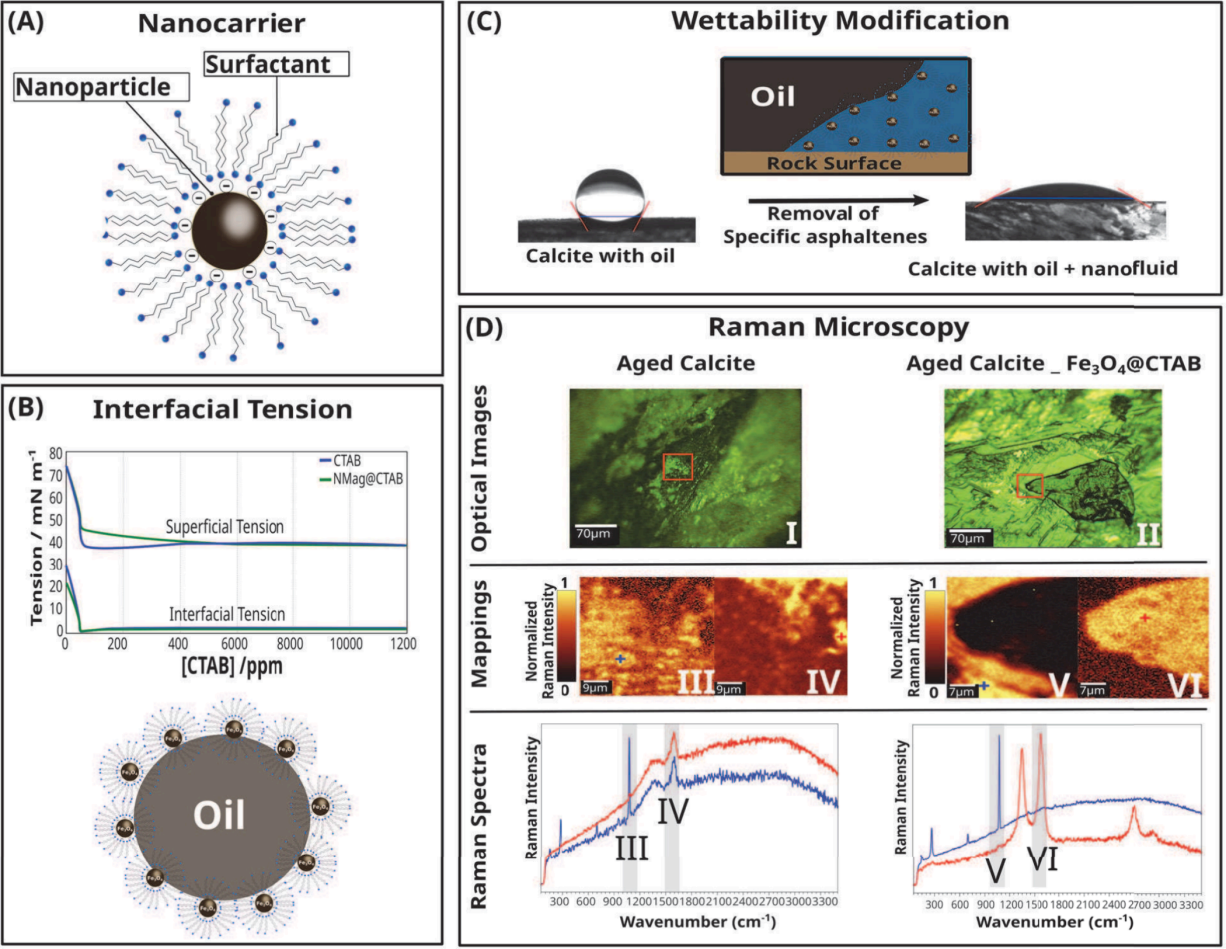
(A) A negatively charged iron nanoparticle serves as a carrier by adsorbing a cationic surfactant, forming a double layer of CTAB on the nanoparticle surface. (B) The nanoparticle system as a surfactant carrier reduces the interfacial tension, as shown by the results. (C) Wettability modification occurs due to disjoining pressure caused by the accumulation of nanoparticles at the oil-rock interface. (D) Raman microscopy data reveal that the mechanism is a result of the extraction of asphaltenes adsorbed on the rock from the oil. Raman mappings (c)/(d) and (e)/(f) display the intensity of the calcite and asphaltene peaks, respectively, of the region marked by the red square on parts (a) and (b). (G) and (h) are the Raman spectra of the points indicated on the last pair of figures. Adapted from reference (38). Licensed under a Creative Commons Attribution (CC BY) license.

Although the mechanism of action of the surfactant-NP combined system is still under debate, we propose some possible mechanisms to explain how these systems enhance oil production. Figure 6 summarizes the main properties altered by these systems, highlighting changes in rock wettability, IFT, and surfactant transport using NPs.^33,194,206,207^ Techniques used to study the mechanisms of action are also shown, including optical microscopy and Raman spectroscopy.

The combination of nanoparticles and surfactants can have different effects depending on their charges. If the surface charge of the nanoparticle is different from the surfactant, two situations can occur. Suppose the rock and nanoparticle have the same charge, and the charge is different from the surfactant. In that case, the surfactant adsorption will be dominant, and the presence of nanoparticles will not influence the IFT between the oil and water phases.^209^ Suppose the rock and the nanoparticle are attracted to the surfactant, but the nanoparticle/surfactant interaction force is stronger than the rock/surfactant. In that case, the system will act as a carrier. The surface coating of the nanoparticle by surfactant will be favored, and the NP@surfactant system will act as a carrier delivering surfactant to the oil/water interface, reducing IFT and surfactant loss by adsorption.^184,207,210^ In systems where the charge of the nanoparticle, rock, and surfactant polar head are equal, there will be activity at the interface due to the electrostatic repulsion. The presence of nanoparticles decreases the IFT because the surfactant will be forced to migrate to the interface. In this case, the nanoparticles do not act as a surfactant carrier, forming a dispersion of nanoparticles and surfactant. The effect on IFT is synergistic.^210,211^

Freitas et al.^177^ investigated mesoporous silica systems as surfactant nanocarriers in EOR to prevent surfactant losses during the process. They used the nonionic surfactant diethanolamide (DEA), obtained from vegetable oil residues, and observed that its adsorption on mesoporous silica surface followed Freundlich isotherms. The silica-DEA systems were found to keep the surfactant adsorbed on the silica surface and release it only at the water–oil interface, reducing the interfacial tension to values below 1mN/m.^177^ In an aqueous medium, the authors observed no surfactant release into the medium. However, in the presence of an oil phase, the surfactant was desorbed from the silica’s surface and migrated to the water–oil interface. This result suggests that the interaction between the DEA-silica system by hydrogen bond is stronger than the interaction of the surfactant with the aqueous phase. However, the hydrophobic interaction of the surfactant’s apolar chain with the oil is more effective when the surfactant reaches the oil–water interface, leading to the system breakdown.^177^

Venancio et al.^178^ used modified silica nanoparticles with alkyl groups to increase the hydrophobic interaction with the surfactant. The authors investigated the interaction mechanisms between these modified NPs and anionic surfactants in nanofluids for EOR. The presence of hydrocarbon chains on the silica surface significantly improved the retention of the anionic surfactant in solution (up to 90%) due to additional hydrophobic interactions with the surfactant tails, which reduced the loss by adsorption. This behavior suggests that this system acted efficiently as a carrier of surfactants, as it limited the amount of free surfactant available for adsorption in the porous medium.^178^

Research that evaluates silica NPs’ behavior in surfactant adsorption in sandstone rocks shows that hydrophilic particles show better results in decreasing surfactant adsorption due to the more significant number of hydroxyl groups exposed on their surface. The particles’ negative surface charge favors their adsorption on the rock surface, which increases the repulsion between the rock surface and the anionic surfactant, decreasing the surfactant loss. Mohammad Ali Ahmadia and colleagues^212^ and Zargartalebi et al.^213^ studied the addition of hydrophilic and hydrophobic silica nanoparticles to evaluate the loss of sodium dodecyl sulfate (SDS). Their studies show that the rate of surfactant loss is directly linked to the concentration of silica used and directly influences the oil recovery rates. Using a theoretical model, Seyyed Shahram Khalilinezhad et al.^214^ proposed that hydrophilic silica nanoparticles and SDS reduce the IFT. This process is related to the repulsive electrostatic forces between the particles and the SDS that cause the surfactant to diffuse until the interface or the nanoparticle’s surface is covered by many surfactant molecules that act as an SDS carrier to the interface.

Pereira et al.^38^ studied the ability of *Fe*_3_*O*_4_ NPs to transport a cationic surfactant, CTAB, and observed a synergistic effect when the system was used to modify the wettability of calcite fragments and to reduce the IFT of oil/water interface. Zeta potential measurements showed that CTAB adsorbed on the negatively charged *Fe*_3_*O*_4_ surface, turning the potential positive by forming a CTBA bilayer. The *Fe*_3_*O*_4_*@CTAB* positive surface charge improved NPs’ mobility through a calcite unconsolidated porous medium during flooding experiments and its stability in brine. This system promoted an increase in oil recovery of 12.8% compared to secondary recovery and a total oil recovery of approximately 60%. Raman and XPS analysis revealed that *Fe*_3_*O*_4_*@CTAB* NPs could remove asphaltene molecules adsorbed on the calcite surface (Figure 6D). The NPs presence improved the wettability modification due to the disjoining pressure exercised during the NPs adsorption on the rock-oil–water interface (Figure 6C). Furthermore, the nanofluid can slow down *CaCO*_3_ scale formation, contributing to the flow assurance during the nanoflooding process. This system proved to be an efficient surfactant carrier in the EOR process.

Ojo et al. introduced an innovative approach to mitigate surfactant loss in enhanced oil recovery.^215^ They utilized clay nanotubes known as halloysites, which have the unique capability to encapsulate surfactants and deliver them precisely to the oil–water interface. Through the creation of nanocomposites comprising Halloysite/Surfactant/Wax, the researchers successfully reduced surfactant adsorption on reservoir rocks, resulting in an impressive 40% increase in oil recovery. This method demonstrates the potential of harnessing nanotechnology to enhance the efficiency of surfactant utilization in the context of oil reservoir management.

Nanoscale particles have unique properties and diverse applications in various technological fields. In EOR, inorganic nanoparticles, primarily silica, have been employed to alter the wettability of oil-wettable rocks and decrease the IFT between oil and water. The ability to modify the surface of these nanoparticles confers great potential for their customization and practical use. Despite this, the mechanisms that assess the efficiency of using nanoparticles as surfactant carriers require further investigation. This approach aims to reduce surfactant losses due to adsorption and precipitation, thereby providing better application conditions for EOR. The main systems applying nanoparticles as surfactant carriers are summarized in Table 2. However, the stability of these systems under high salinity conditions and the effects of different nanoparticle geometries and sizes need to be explored more comprehensively. There is a lack of experimental studies showing the relationship between particle shape and disjoining pressure, wettability inversion, and oil recovery factor. These factors are of utmost importance to avoid nanofluid stability issues that can obstruct the pore throat and compromise well integrity, rendering the use of nanofluids in EOR.

**Table 2:** Inorganic Nanoparticles as Surfactant Carriers

Carrier	Surfactant	Mechanism	Ref
Mesoporous *SiO*_2_	DEA	DEA adsorption on the mesopourous, and release only into the oil–water interface, reducing interfacial tension.	(177)
*SiO*_2_ NPs	SDS	Formation of highly charged nanoclusters between the alkyl-modified nanoparticles and the anionic surfactant. Reduced the loss by adsorption and reduction of IFT.	(178), (213)
*ZrO*_2_ NPs	SDS, CTAB	Reduction of the interfacial tension due to the increased surface activity of surfactants in the presence of nanoparticles.	(216)
*SiO*_2_ NPs	CTAB	1 - Analysis of the synergy effect on the reduction of interfacial tension attributed to the competitive adsorption of silica particles and CTAB molecules at the oil–water interface applied to the stability of emulsions. 2 - CTAB-silica nanoparticle interaction and its complexes are elucidated by dynamic IFT data and elasticity measurements.	(209), (217)
*Al*_2_*O*_3_, *ZrO*_2_, , *TiO*_2_	Short chain carboxylic acids, alkyl gallates, and alkylamines	Stabilization of Foams and IFT decrease lead to a decrease of the air–water interface area when surfactants were added above the CMC.	(218), (219)
Hydrophobic *SiO*_2_ NP	CTAB, SDBS	The evaluation of the surface tension and reduction of interfacial tension of the NP/surfactant mixture by zeta potential suggests that nanoparticles interact with surfactants in competition with the air–water surface and oil–water interface.	(220), (221)
*Fe*_3_*O*_4_ NPs	CTAB	High surfactant load. The NPs can remove adsorbed asphaltes from the rock surface. Reduced the loss by adsorption and reduction of IFT.	(38)

### 3.2 Carbon Nanomaterials

Carbon-based nanomaterials, such as carbon nanotubes and graphene and its derivatives, have unique properties that make them promising candidates for various applications, including optoelectronics,^222^ sensors,^223,224^ energy storage and conversion,^225^ flexible electronic devices,^226−228^ biomedical,^229,230^ and oil and gas operations, including EOR.^173,231−234^

Synthetic pathways for carbon nanotubes, graphene, and their derivatives, which aim to attain specific characteristics such as size, layer count, defects type and density, and atomically sharp edges, encompass a spectrum of methodologies. These include top-down approaches, such as electron beam lithography,^235^ nanoimprinting, scanning probe lithography,^236^ and liquid or chemical exfoliation,^237−239^ as well as bottom-up methods involving chemical vapor deposition,^240,241^ surface-assisted chemical reactions, and conventional organic reactions.^242^ These diverse strategies hold promising potential for achieving precise control over both size and shape, as well as the deliberate engineering of defects and edges.

The interaction between the surface of carbon materials and surfactants can occur via pi-stacking or through interactions with surface functional groups or heteroatoms. Hence, understanding the role of synthesis control is crucial, as it has the potential to determine the chemical properties of graphene. In particular, chemical vapor deposition growth in N or O-rich environments facilitates the generation of materials with heteroatoms.^243,244^ Conversely, chemical exfoliation in a highly oxidative medium introduces multiple functional groups, such as epoxy, hydroxyl, and carboxyl.^245,246^ Additional synthesis techniques can induce point defects, including single and double vacancies, thereby creating multiple interaction sites.^247,248^ This rich diversity opens up numerous possibilities for modeling new graphene materials for surfactant carrier systems. Further insights into the synthesis of carbon materials can be gleaned from comprehensive reviews available in the literature.^237,241,245^

Considering EOR applications, Chen et al.^36^ studied multiwalled carbon nanotubes (MWNT) as surfactant carriers in EOR. The strong affinity of MWNTs with carbon black systems’ hydrophobic tails enables them to carry a high density of surfactants. Competitive surface adsorption of the surfactant against the rock surface reduces the adsorption loss of α-olefin sulfonate at concentrations below the CMC. The results of the microemulsion phase behavior confirmed that the MWCNTs successfully and spontaneously released the surfactant at the oil/water interface once they came into contact with the oil. The presence of these nanotubes did not influence the ultralow oil/water IFT values, which were measured around 0.007–0.009 mN/m. The utilization of carbon nanotubes in surfactant injection presents a breakthrough by facilitating selective permeability, enhancing effectiveness, and mitigating the drawbacks associated with injecting isolated surfactants. Furthermore, these carbon nanotubes play a pivotal role in stabilizing emulsions, contributing to the overall efficiency and success of the injection process in enhanced oil recovery.^46^

The development of carbon nanotubes is an expensive method and requires thorough documentation for application in EOR. However, in pursuit of a more sustainable and environmentally friendly approach that leverages technological advancements in new materials, Bashirul Haq et al. developed carbon nanopartilces from date-leaf biomass (DLCNP) as a green fluid injection. An 800 ppm sample of DLCNP was mixed with 0.5 wt% of the green nonionic surfactant Alkyl Polyglucoside (APG) and 2 wt% NaCl brine. This formulation achieved 45% tertiary oil recovery and 89% original oil in place (OOIP) recovery in sandstone formations, outperforming commercially available carbon nanotubes. These results confirm the efficiency of DLCNP as a surfactant carrier in EOR applications.^249^

Graphene and its derivatives, such as graphene oxide (GO), are two-dimensional carbon nanomaterials with a large specific surface area, high thermal conductivity, and mechanical strength^233,250,251^ that hold great potential for developing nanofluids for EOR.^173,234^ The chemical modification of graphite through the oxidation process and defect creation allows the creation of negative charges on the GO’s surface caused by the presence of oxygen functional groups.^245,252^ GO is a candidate for EOR due to its superhydrophilicity and superhydrophobicity from its functional groups and the graphene-like basal plane, respectively.^233,253^ Such behavior positively impacts rock wettability, oil/water IFT, and emulsion stability. Dinesh Joshi et al.’s experimental research demonstrates that the nanofluid composed solely of graphene oxide nanosheets in an aqueous solution induces a notable alteration in the wettability of sandstone reservoir rock. This transformation is evidenced by a substantial reduction in the contact angle, decreasing from 112.4 to 17.2°. Additionally, the introduction of graphene oxide nanosheets results in a decrease in the interfacial tension between the oil phase (decane) and the aqueous phase, dropping from 42.34 to 32.76 mN/m. Notably, the presence of these nanosheets leads to the emulsification of crude oil, representing a crucial mechanism in EOR.^10^ However, real well conditions, such as high salinity, will influence the stability of these systems.

The stability of GO nanofluids presents itself as a major challenge for EOR, considering that the instability of the suspensions can cause agglomeration of particles and problems such as pores clogging and reduction of the reservoir relative permeability. GO nanofluids stability is due to the electrostatic repulsion between the negatively charged oxygen functions on the nanosheet surface and translates into greater resistance to aggregation.^173,254,255^ However, this system can be affected by the concentration of electrolytes, the nanofluid concentration, and the pH.^256^ The stability challenge becomes even greater in high salinity environments, where there is a drastic reduction in GO’s electric double layer thickness due to strong ionic interactions.^256,257^

The oxidation degree, nanosheet size and thickness, nanofluid concentration, and the presence of surfactants are key factors that influence GO nanofluid stability.^252^ There are several strategies to improve the stability of GO suspensions, among which its mixtures with surfactants and polymers stand out.^173,257^ Graphene oxide, chemically stabilized by polymers, plays a dual role in nanofluid dynamics. First, it contributes to enhancing the viscosity of the nanofluid in comparison to utilizing an isolated polymer. Second, owing to its sturdy structure, the nanofluid has the capability to decrease the interfacial tension between oil and water. This property, in turn, induces a shift in wettability, transitioning the surface of sandstone slices from oil-wet to water-wet. The combination of these effects showcases the versatile impact of graphene oxide-polymer stabilization in altering the properties of the nanofluid for potential applications, particularly in scenarios like EOR.^258,259^

**Figure 7:**
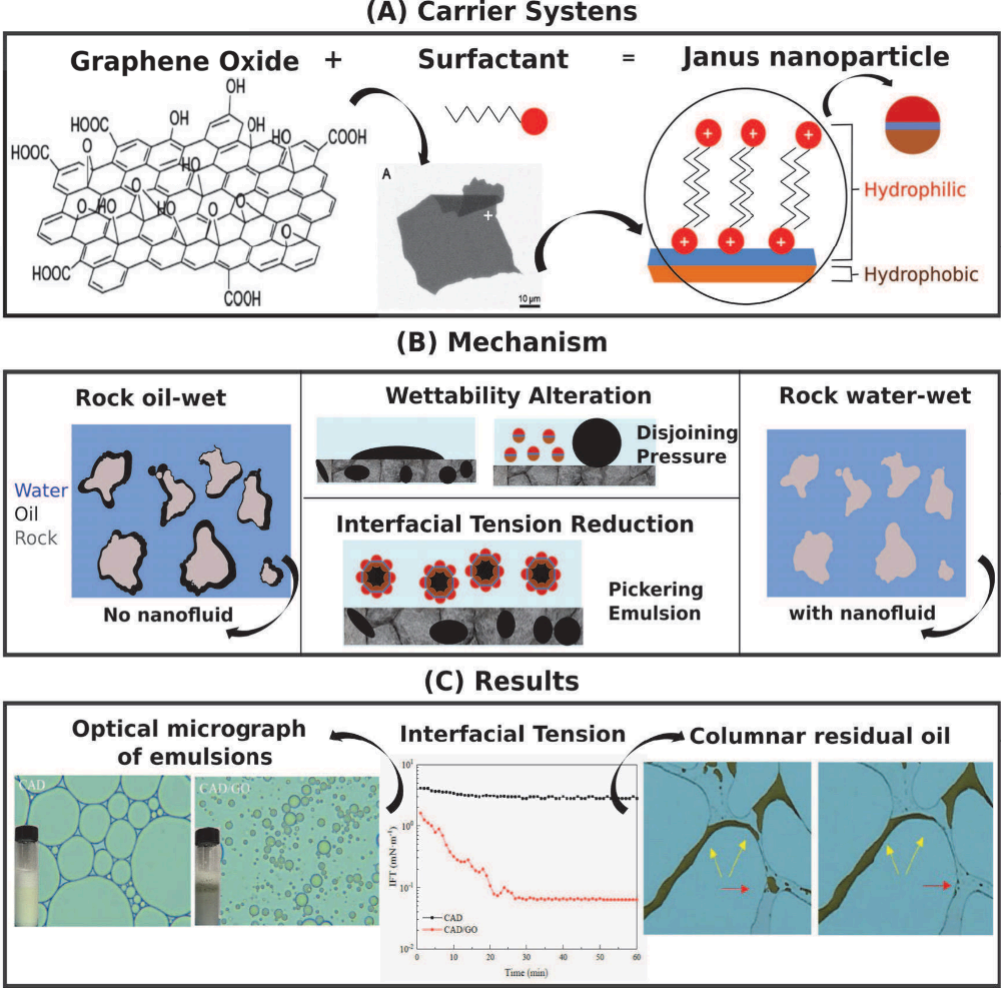
(A) A carrier system is formed by the interaction between cationic surfactant and graphene oxide (GO) nanosheets to create Janus nanosheets. (B) The system alters the wettability of oil-wet rock through two mechanisms: the inversion of wettability due to disjoining pressure and the formation of a Pickering emulsion. (C) The particle size of the emulsion formed by the CAD/GO (amphoteric surfactant disodium cocoamphodiacetate/graphene oxide) dispersion system is smaller than that of the emulsion formed by CAD aqueous solution. The oil/water interfacial tension of CAD aqueous solution and CAD/GO is reduced. A reduction in residual oil saturation is observed, as shown by the gradual deformation and removal of the columnar residual oil front edge by the displacement fluid, leading to improved displacement efficiency. Techniques such as microscopy and Raman spectroscopy are used to study the action mechanism. Adapted with permission from ref (234). Copyright 2022 Elsevier.

Another possibility is surface modification through the synthesis of Janus materials, whose main characteristic is the spatial organization of the charges.^260−262^ Janus nanosheets exhibit constrained rotation at the fluid-fluid interface, leading to enhanced and oriented adsorption at this interface.^263−265^ In this system, one face of the GO sheet is hydrophilic, while the other face is hydrophobic. The hydrophobic segment usually comprises the graphene-like basal plane or surfactant adsorbed on the GO surface, where the nanosheets act as surfactant carriers.^266^ Despite the good results regarding the stability of GO nanosheets using these strategies, there are still few studies in this area, which makes it difficult to understand their role in the EOR process. Figure 7 illustrates the potential action of GO Janus nanosheets in the oil reservoir. Its special arrangement favors the modification of rock wettability, stabilization of oil droplets, and reduction of interfacial tension.^266^

Luo et al.^262^ investigated nanofluids consisting of low concentrations of graphene-based Janus amphiphilic nanosheets for EOR. The study demonstrated a 15.2% increase in the oil recovery factor. Stability tests indicated that the nanosheets accumulated at the oil/water interface, forming a film that acted like an interfacial elastic layer. The authors attribute these characteristics to the high oil recovery efficiency.

Radnia et al.^257^ studied a new sulfonated graphene-based nanofluid for EOR processes. Functionalizing the GO sheets increased the colloidal dispersion’s stability, with a zeta potential of −30 mV, and reduced its adsorption on the rock. The material stabilized oil/water emulsions, maintaining oil droplet size between 1 and 3 μm and reducing the oil/water interfacial tension by 12%. The authors suggest that this material’s main mechanism of action is the change in wettability, which increased the oil recovery factor by up to 19% in oil displacement tests in carbonate porous media. A related study conducted by Jie Cao and colleagues demonstrated the successful large-scale synthesis of Janus sulfonated graphene oxide nanosheets. The outcomes revealed that these systems exhibited a remarkable 71% reduction in interfacial tension and achieved an additional recovery factor of 18.4%.^267^

Figure 7 illustrates the performance of these systems inside the reservoir, and Table 3 summarize their mechanism of action, advantage, and disadvantages. Carbon materials, including graphene and its derivatives and carbon nanotubes, are the most promising surfactant carriers due to their ability to alter rock wettability and accumulate nanosheets at the oil–water interface, generating elastic interfacial films. However, the understanding of their interaction with surfactants and their performance as surfactant carriers is still limited, and their stability in high salinity, pressure, and temperature environments presents a challenge. Therefore, a thorough investigation of the formation of Janus nanosheets through the interaction of GO with surfactants is essential to understand their behavior and performance in EOR processes. This approach could pave the way for the development of new nanofluids with high application potential as EOR additives, ultimately increasing the profits of the oil and gas industry.

**Table 3:** Carbon Nanomaterials as Surfactant Carriers

Carrier	Surfactant	Mechanism	Advantage	Disadvantage	Ref
Janus GO with dodecylamine	DTAB SDS TRITON X-100	IFT reduction and decrease of asphaltene precipitation	Ultralow IFT (*IFT* = 8 × 10^3^ mN/m)	Complex synthetic route	(261)
MWNT	anionic surfactant alpha olefin sulfonate (AOS)	spontaneously surfactant release to the oil/water interface	ultralow oil/water IFT (0.007–0.009 mN/m). MWNTs high surfactants load.	NPs retention blocking pore throats, decrease of rock permeability	(36), (46)
Janus GO with Alkylamine	sodium dodecyl sulfate (SDS) and TWEEN 20	(i) the accumulation of nanosheets at the oil/water interface, (ii) the appearance of climbing films, and (iii)the generation of elastic interfacial films	High performance at low concentration. Good stability in brine	Complex synthetic route	(262)
Sulfonated graphene		Potential surfactant carrier due their structure	Wettability alteration		(257)

### 3.3 Polymers

Polymer injection is a chemical EOR method that aims to improve the reservoir sweep efficiency by reducing the mobility ratio and decrease the EOR operational costs by reducing the volume of water injected. Viscosity is a key parameter in the EOR process that can increase the capillary number and decrease the mobility ratio, as showed in Equation 1. The viscosity of the injected water can be increased by adding polymers, which can also decrease the relative permeability to water in some cases.^48,268,269^

For a good polymeric EOR agent, it is important to consider its chemical, thermal, mechanical, and biological stabilities, and a good viscosifying power is obviously important. The presence of R-O-R groups should be avoided due to their low thermal stability,^270^ and the presence of ionic groups should be carefully planned, similar to surfactants. Originally, anionic polymers like Partially Hydrolyzed Polyacrylamide (HPAM) were exclusively employed in sandstone reservoirs to mitigate adsorption issues.^268^ Nevertheless, owing to the potential for structural modifications, numerous projects are now extending the application of these polymers to carbonate rocks. Nonionic hydrophilic groups improve the chemical stability of the material.^7,270^ The stability of the polymer is affected by its concentration, temperature, salinity, and pH of the aqueous medium.^3,7^

In addition to the polymer’s characteristics, their rheological properties are also important, as they can improve the sweeping efficiency, retention rate, pore accessibility, and permeability. Therefore, it is important to carefully study the behavior of the polymer inflow during the elaboration of an EOR project.^7,271^

The polymers used in EOR can be categorized according to their origin, with synthetic, natural or bio polymers being the main groups. HPAM is the most commonly used synthetic polymer for EOR flooding, while xanthan gum, guar gum, cellulose, and chitosan derivatives are the most used biopolymers in EOR.^4,7^ Synthetic polymers are synthesized through various routes, including addition or condensation reactions. In contrast, natural polymers are sourced from microorganisms and plants, with the extraction method influencing characteristics such as molecular weight and size. The size of polymeric structures is a crucial property that influences the carrier’s action mechanism, which is determined during the synthesis process. Consequently, dendrimers and hyperbranched polymers present intriguing options for carriers compared to linear polymers due to their unique structural properties.

When injected alongside surfactants in the SP method, the presence of both polymer and surfactant alters the solutions’ rheological properties, adsorption characteristics at solid–liquid interfaces, stability of colloidal dispersions, liquid–liquid interfacial tensions, among other physicochemical properties.^272^ Therefore, it is important to understand the interactions between polymer and surfactant, which can be relatively weak interactions between polymer chains and surfactant head groups or strong electrostatic interactions between oppositely charged polyelectrolytes and surfactant head groups. Hydrophobic interactions between the polymer and surfactant chains can also occur, and in some systems, they are the predominant interaction forces.^273−275^

**Figure 8:**
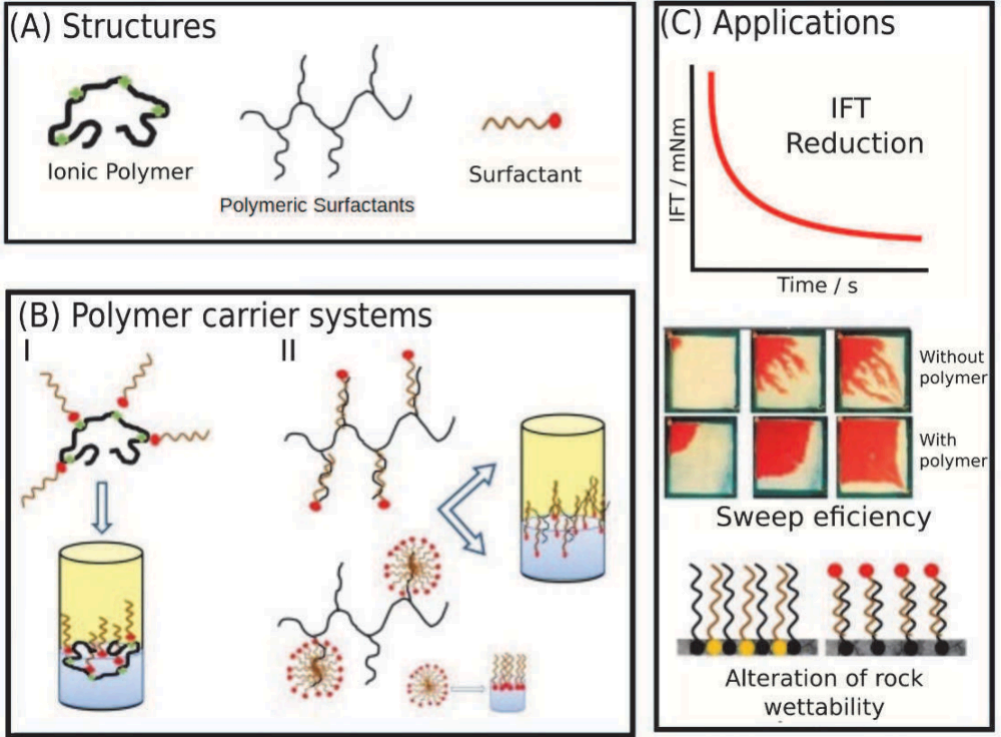
(A) Polymers have high potential as surfactant carriers. (B) The polymer carrier system operates through electrostatic interactions between a cationic polymer and a nonionic or anionic surfactant. Ilustartion of a polymeric surfactant carrier at the oil–water interface (I). Another type of polymer-surfactant interaction occurs between the hydrophobic polymer and the surfactant through tail–tail interactions, as shown in (II). The hydrophobic polymer can also interact with the inside of the micelles. These systems are illustrated at the oil–water interface. (C) These polymer-surfactant systems can alter interfacial properties such as IFT, sweep efficiency, and rock wettability.

There is a tendency to assume that hydrophobic modifications in polymers strengthen the polymer-surfactant interaction, providing hydrophobic sites to which nonionic surfactants preferentially bind. The competition between the self-assembly of hydrophobic polymers, the surfactant micellization process, and the hydrophobic interactions between polymers and surfactants is a characteristic of polymer-surfactant systems.^275,276^Figure 8 illustrates the possible interactions between polymers and surfactants, demonstrating that the presence of surfactants can cause changes in the polymeric chain’s conformation and in the system’s effective size. Polymer-surfactant interactions can occur at the molecular level and between micelles and/or polymer aggregates, as shown in Figure 8B.^272^ This way, polymers can act as carriers for individual surfactant molecules or micelles, delivering them at the water/oil interface.

Recent studies have addressed the importance of physicochemical interactions in surfactant-polysaccharide systems, highlighting the structures formed in solution by inter- and intramolecular interactions.^277^ The characteristics of the solutions can be controlled through the polysaccharide structure, either through the presence of charges or hydrocarbon segments, which will provide electrostatic or hydrophobic interactions, respectively.^277^ While the interaction between polymers and ionic surfactants is widely studied, the interaction between polymers and nonionic surfactants is less explored and occurs preferentially through hydrophobic associations.^276^ For instance, Nagarajan et al. found that the complexation between polyvinyl pyrrolidone and polyethylene oxide surfactant Triton X-100 was not favored due to the formation of free micelles instead of polymer-surfactant complexation.^272^

Using particle size and zeta potential measurements, Ofridam et al. investigated the interaction between nonionic surfactant Triton-X-100 and polymethylacrylate derivatives (anionic and cationic). They observed no interaction between Triton X-100 and these ionic polymers due to the favored intramolecular interaction over intermolecular polymer-surfactant interactions. In contrast, the interaction between the ionic surfactants and the polymers of opposite charge resulted in precipitates due to electrostatic interactions.^278^

Research has shown that biopolymers such as chitosan, cellulose, and their derivatives interact with both ionic and nonionic surfactants. Grant et al. investigated the intermolecular interactions, morphology, and associations between mixtures of cationic chitosan and nonionic sorbitan esters. They used measurements of surface tension, turbidity, and conductivity to show that the size and architecture of these systems depend on the structure and concentration of the surfactant used. The association mechanism between chitosan, surfactant, and chitosan-surfactant aggregates is related to the rigidity and hydrophobicity of the species.^279−281^

dos Santos Francisco et al. examined cationized chitosan (TMC) as a carrier for the surfactant oleic acid diethanolamide (OADA) in the context of EOR.^282^ The evaluation of TMC’s interactions with OADA and its ability to store the surfactant involved a comprehensive analysis covering IFT measurements, particle size characterization using dynamic light scattering (DLS), and molecular dynamics (MD) simulations. The results revealed that TMC exhibited a planar conformation in water/salt solution and in a heptane/toluene mixture (Heptol). Furthermore, TMC demonstrated the ability to interact with the surfactant, forming a complex suitable for acting as a surfactant carrier with a size compatible with permeating reservoir pores. In a water/salt solution, the TMC-OADA complex consisted of OADA micelles interacting with the TMC surface.^282^ IFT measurements showed that the presence of TMC accelerated the reduction of IFT compared to OADA alone, demonstrating its significant potential for EOR applications by achieving ultralow IFT values of 10^–2^ mN/m. MD simulations further revealed that the TMC chitosan derivative maintained the polymer/surfactant complex during diffusion through the water/salt medium and released the surfactant only at the water/oil interface. Additionally, once the surfactant migrated to the oil phase, the chitosan derivative-OADA complexes did not reform, minimizing adsorption losses during the injection phase of the EOR process. Experimental and theoretical findings collectively highlighted the performance of TMC as an OADA surfactant carrier, underscoring its potential for polymer/surfactant flooding in EOR applications. Therefore, chitosan-surfactant systems possess some of the necessary characteristics of a carrier for EOR applications.

Another approach involves utilizing polymer emulsification reactions to incorporate surfactants into specific structures such as organic nanoparticles or lipid matrices. Organic nanoparticles derived from polymers, such as polystyrene and lipid matrices, have been investigated as surfactant carriers. For instance, Avila et al. synthesized polystyrene nanoparticles as surfactant carriers,^35^ while Caplan et al. synthesized sulfonated polystyrene nanoparticles for the same purpose.^37^ In both cases, the surfactant was absorbed inside the nanoparticles during the formation of the nanoparticle. These systems exhibited ultralow interfacial tension and could inhibit the adsorption of the surfactant on the surface of sand particles. Additionally, these systems allowed the controlled release of surfactant upon contact with the oil phase. Polystyrene nanoparticles have a hydrophobic matrix, leading to a nonpolar character in the inner part of the nanoparticle, which allows the surfactant storage due to tail-tail interactions. However, when this system comes into contact with the oil phase, new hydrophobic interactions occur between the nanoparticle matrix and the oil phase, causing the polymer to swell and replace surfactant-nanoparticle interactions with oil-nanoparticle interactions, releasing the surfactant and reducing surfactant losses during the transport test, which caused an increase in oil recovery.^35,37^

Rosestolato et al. showed that organic nanoparticles formed by a lipid matrix could store surfactant in their core due to hydrophobic interactions, releasing the surfactant only on the oil interface. These nanocarriers exhibited 96% storage capacity of surfactant and high mobility in porous media. Lipid nanoparticles are highly stable systems capable of reducing interfacial tension to ultralow values, emphasizing the great potential of this system as a surfactant nanocarrier for the EOR process.^39^

The use of polymers and organic nanoparticles as surfactant carriers is an interesting strategy that can improve both sweeping and displacement efficiency. Polymers not only transport the surfactant but also increase the system’s viscosity. Therefore, hydrophobic interactions between the polymers and surfactants are crucial for the system to function correctly. With regard to organic nanoparticles, they operate on the principle of swelling in the presence of oil, which releases the surfactant. Additionally, they may alter rock wettability through disjoining pressure, although this property needs to be further investigated.

In general, polymers intended for use as carriers need to undergo chemical modification to enhance their interaction with surfactants. Typically, these modifications involve grafting reactions to promote hydrophobic association.^283^ In this context, this review considers it important to address a subclassification of polymers, called polymeric surfactants, which demonstrate a great ability to act as carriers for surfactants applied in EOR methods.

#### 3.3.1 Polymeric Surfactants

Polymeric surfactants are macromolecules composed of both hydrophobic and hydrophilic groups. Due to their unique structural characteristics and surface and interfacial properties, they are technically considered surfactants. Previously, they were referred to as hydrophobically modified polymers or amphiphilic polymers. For clarity and consistency in this review, the term “polymeric surfactant” will be used to focus on their role as surfactant carriers in EOR.^284−286^

The application of polymeric surfactants in EOR has been extensively documented by various authors. These surfactants offer several advantages, including economic benefits, effective mobility control, and the ability to reduce interfacial tension, thereby improving oil recovery by increasing the fractional flow of oil.^284^ They also have significant potential for altering the wettability of oil-wet rocks. A thorough understanding of the mechanisms of these additives as surfactant carriers is crucial, as they influence the CMC, interfacial tension, and wettability.^287,288^ In addition to their promising applications in EOR, polymeric surfactants can reduce the viscosity of heavy oils, act as carriers, and facilitate the formation of emulsions.^284^

Although high molecular weight polymeric surfactants exhibit surface activity, they do not achieve the ultralow IFT values required for effective oil displacement in EOR applications.^289^ A literature review by Funsho Afolabi et al. discusses the use of polymeric surfactants for EOR, noting that only a few studies report moderate success in reducing surface and interfacial tensions.^284^ However, polymeric surfactants can alter the wettability of oil-wet rocks, thereby increasing oil production rates through surface adsorption or cleaning mechanisms. This wettability alteration has been highlighted by several authors.^290−292^

For example, El-Hoshoudy et al. used hydrophobically associating polyacrylamide (HAPAM) in saline solution.^293^ Kumar et al.,^294^ Babu et al.,^295^ and Wibowo et al.^296^ investigated the wettabillity alteration capacity of polymethyl ester sulfonate (PMES), an anionic polymeric surfactant, in sandstone. Bai et al. studied hydrophobized hydroxyethyl cellulose.^297^ All these studies concluded that wettability alteration occurs through adsorption mechanisms and is influenced by factors such as time, polymeric surfactant concentration, and brine salinity.

Considering the benefits and drawbacks of using polymeric surfactants and commercial surfactants in EOR, a thorough cost-benefit evaluation is necessary. Additionally, understanding the physicochemical processes involved in rock-oil-fluid interactions is crucial. Recently, interactions between polymeric surfactants and conventional surfactants have been extensively studied due to their relevance in various industrial applications, including coatings,^298^ oil recovery,^299,300^ cosmetics,^301,302^ food,^303^ and pharmaceuticals.^304,305^

This section of the review presents studies that explore the combination of polymeric surfactants with conventional surfactants and how this associative system can serve as a potential surfactant-carrying mechanism in EOR. One of the first water-soluble polymer/surfactant systems studied was poly(ethylene oxide) with SDS.^306^ The presence of surfactant affects the rheological properties of the polymeric surfactant, such as viscosity and molecular conformation. Conversely, the presence of polymers with some degree of hydrophobicity alters the behavior of aqueous surfactant solutions. S. Biggs, J. Selb, and F. Candau showed that hydrophobically modified polyacrylamide in SDS solution exhibited two critical concentrations of surfactant, both different from the CMC of pure surfactant.^307^ This occurs because hydrophobic chains induce the aggregation of surfactant micelles at a concentration below the CMC, called the critical aggregation concentration (CAC). Once all available polymeric sites for interaction are saturated, adding more surfactant leads to the formation of free micelles in solution above the CMC.

Studies of interactions between surfactants and various polymer-surfactant systems, such as hydrophobically modified hydroxyethyl cellulose, poly(ethylene oxide), polyacrylamides, poly(acrylic acid) and their copolymers, as well as thermosensitive polymers like poly(N,N-diethylacrylamide) and poly(N-isopropylacrylamide), have been extensively developed. These interactions have been evaluated using various methods, including fluorescence techniques.

A notable study conducted by L. Piculell et al. evaluated the properties of mixed solutions of surfactants and hydrophobically modified polymers (polymeric surfactants), with a focus on molecular aspects such as self-assembly, mixed aggregation, phase behavior, rheology, and interfacial behavior.^308^ They demonstrated that the presence of hydrophobic groups enables these structures to self-associate through the formation of extensive noncovalent networks, resulting in micelle-like aggregates. This network produces a significant thickening effect, which is one of the most technologically important properties of polymeric surfactants. The authors emphasized that a diblock copolymer modified at only one end cannot function as an associative thickener and will act as a traditional surfactant instead. The presence of hydrophobic groups also increases miscibility with anionic surfactants, such as SDS, as mixed aggregates form. The surfactant associates with the polymeric surfactant both in bulk solution and at interfaces, or competes for the silicate surface. The presence of surfactant can reduce polymeric adsorption in two ways. One interpretation suggests that the association of aggregates creates a complex more soluble than individual additives, minimizing adsorption and allowing the polymeric surfactant to act as a surfactant carrier. Another way is through competition, where the mixed aggregate breaks apart, resulting in adsorption of a sacrificial agent rather than a carrier system. The interfacial behavior of these aggregates (polymeric surfactant–surfactant) was also evaluated, though the authors noted it is very complex due to the interaction of two surfactant agents.

Another study by Paula Relógio et al. developed copolymers grafted with hydrophobic dodecyl groups and fluorescent dyes such as pyrene, phenanthrene, or anthracene. The interaction of these polymers with an anionic surfactant SDS, was studied using fluorescence techniques. These studies provided direct evidence of the formation of intermolecular aggregates, as well as insights into the number and size of these aggregates after the addition of SDS. Similarly, the interaction between SDS and partially hydrolyzed hydrophobically associating polyacrylamides in different polymer concentration regimes was investigated through measurements involving rheology, fluorescence, conductivity, and zeta potential.^309^

Another finding from the study conducted by Shaohua Chen et al. revealed that the introduction of the HPAM polymer did not alter the IFT of the nonionic surfactant dodecylglucopyranoside. Notably, solutions of HPAM with sodium dodecyl sulfate and HPAM with dodecyltrimethylammonium bromide exhibited lower IFT compared to pure surfactant solutions. These outcomes affirm that the electrostatic interaction between the polymer and ionic surfactants caused a modification in the arrangement of surfactant molecules at the oil–water interface.^274^

Desbrieres et al. studied the interfacial activity of chitin and chitosan in the presence of surfactants and observed an improvement in the surface activity of ionic chitosan derivatives when an oppositely charged surfactant was added. This surfactant acted as a counterion, forming a polyelectrolyte surfactant complex, which was more efficient than alkyl derivatives. The concentration of surfactant required to achieve the desired properties was much lower than its critical micellar concentration in pure solution.^280,310−312^

Perez-Gramatges et al. studied the interactions between the hydrophobized (TMC-C14) and cationized (TMC) chitosan derivatives and the nonionic surfactant NF-10 (nonylphenol) using interfacial tension measurements. They demonstrated that the modified polysaccharide-surfactant systems showed synergy in reducing the IFT, reaching values below 1 mN/m in a saline medium. The authors discussed the importance of the hydrocarbon segment in the hydrophobized chitosan derivative responsible for hydrophobic associations with the NF-10 surfactant. They suggested that these properties may be beneficial in EOR applications, as the molecular arrangement enhances the carrier role.^313^

The studies described by dos Santos Francisco et al. also evaluated TMC-C14 as a carrier for OADA for use in EOR, using IFT, DLS, and MD measurements.^282^ MD results revealed that the solvent and the presence of the hydrophobic segment in the chitosan structure altered the conformation of the structure. In a water/saline solution, the structure presented an entangled conformation and a planar conformation in Heptol. In a water/salt solution, the TMC-C14-OADA complex involved interactions between hydrocarbon segments. This complex was able to store a greater amount of surfactant and also act as a surfactant carrier with a size compatible with permeating reservoir pores. Similar to the TMC-OADA, the TMC-C14-OADA complex achieved ultralow IFT values of 10–2 mN/m and remained combined during diffusion until reaching the interface. Thus, this study highlighted the excellent performance of TMC-C14 as an OADA surfactant carrier, underscoring its potential for combined injection with surfactant in EOR.^282^

The defining characteristic of polymeric surfactants is their simultaneous possession of hydrophobic and hydrophilic groups. In addition to linear polymeric surfactants, hyperbranched polymers hydrophobized with polyglycerol-based structures also function as polymeric surfactants due to their three-dimensional structure and numerous functional terminal groups. These hyperbranched polymers are versatile, offering biocompatibility and the ability to encapsulate and release materials. They are also effective at reducing interfacial tension and reversing the wettability of oil-wet rocks.^298,314^

In this perspective, a hyperbranched polymer derived from polyglycerol (HPG) was evaluated by Ferreira et al. as a carrier for cationic surfactant CTAB in sandstone or carbonate reservoirs. The innovation of this study was to evaluate the storage capacity of the cationic surfactant in the HPG polymer core. The polymer’s size and molecular arrangement allowed the permeation along the porous medium. The authors demonstrated the system’s EOR potential through measurements of interfacial tension and contact angle and the formation of the HPG-CTAB complex by CMC changes, particle size, and zeta potential. The HPG-CTAB recovery factor was found to be higher than that of pure surfactant in both mineralogies.^172^

### 3.4 Supramolecular Systems

Supramolecular surfactant carriers are based on macromolecules that can form host–guest complexes with surfactants. The small size of the host–guest complex and the high number of functional groups are characteristics that enable permeation in the porous medium and reduce surfactant losses.

**Figure 9:**
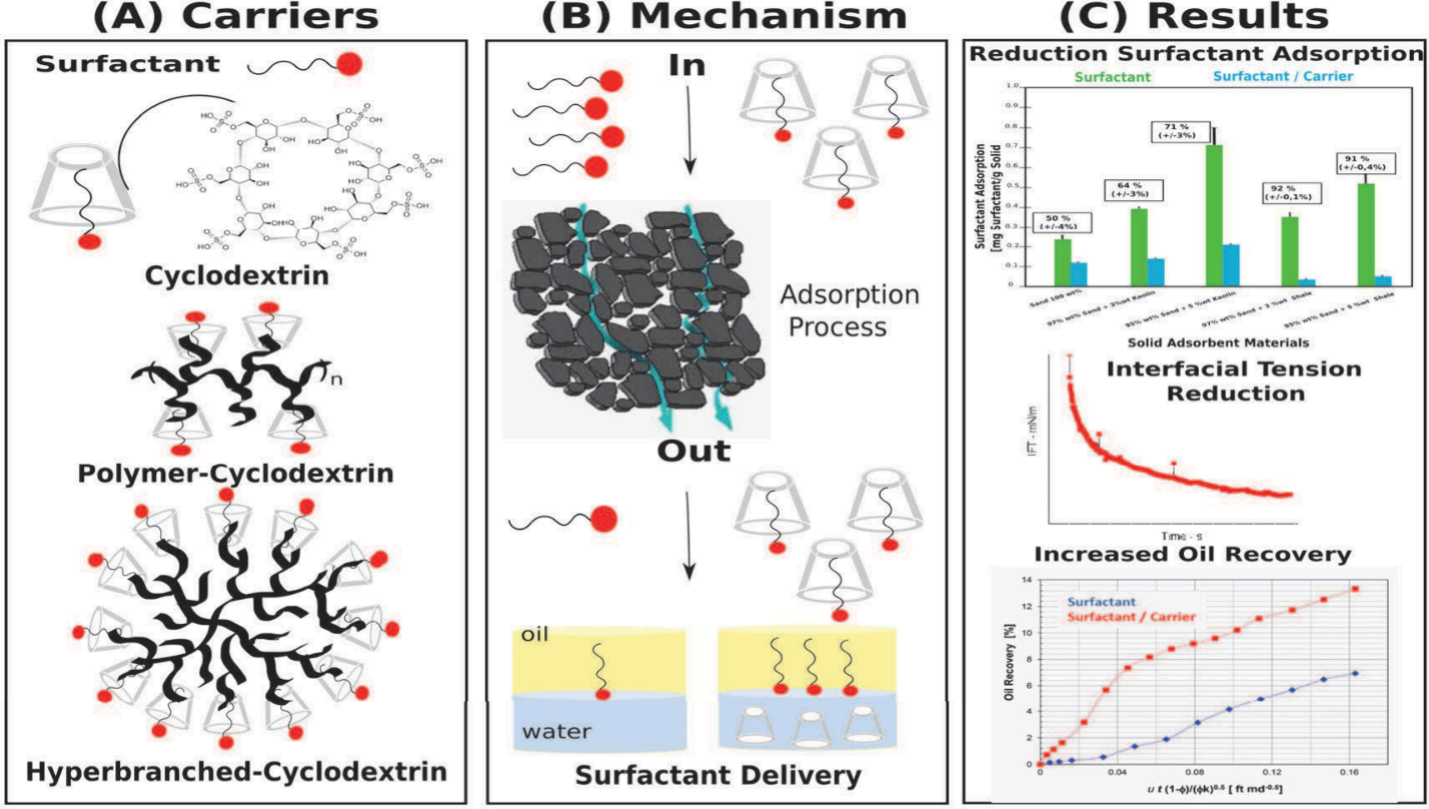
(A) b-Cyclodextrin and its derivatives can act as surfactant carriers, either as complexes with surfactants or as part of polymer or hyperbranched structures. (B) Illustrates the process of surfactant flooding in porous media, comparing the absence and presence of b-cyclodextrin and its derivatives as surfactant carriers. Adsorption processes are also shown in both cases. (C) The main properties changed by b-cyclodextrin and its derivatives include reduced surfactant adsorption, reduced interfacial tension, and increased oil recovery. Adapted with permision from reference (170). Copyright 2015 Canadian Society of Chemical Engineering.

Cyclodextrin (CD) and its derivatives, prepared by enzymatic treatment of starch, have been used in several areas of the oil industry, including EOR. Guest–host systems involving CD and surfactants can work as carrier or delivery systems of surfactants; the host–guest interaction increases the surfactant’s CMC and solubility, effectively reducing surfactant adsorption on rock formations and facilitating surfactant release only in oil saturation zones. Alhassawi et al. used the hydrophobic cavity of β-cyclodextrin as a complexation zone for surfactant solutions through hydrophobic interactions, enabling its transport to the oil area (Figure 9A). This surfactant is trapped inside CD’s hydrophobic cavity, hindering its adsorption on the rock surface.^169,170,315^

Tang et al. reviewed the recent development of cyclodextrin-based materials used in oilfield applications, observing that CD and its derivatives were applied in three different forms in the EOR process: cyclodextrin-based polymers, complexation of surfactant/β-cyclodextrin, and cyclodextrin-based surfactant/polymer binary systems.^169,170,315,316^Figure 9 provides an overview of CD and its derivatives structures that can act as surfactant carriers, proposing a mechanism of action and highlighting the improvements achieved using polymer and hyperbranched derivatives in these systems. The main properties affected by these carriers include a reduction in surfactant adsorption, a decrease in IFT, and an increase in oil recovery.

Alhassawi’s experimental results demonstrated that SDS/β-CD complexes outperformed conventional surfactant flooding regarding oil displacement capability. Dynamic adsorption data confirmed the effectiveness of host–guest complexes in reducing surfactant adsorption on solid surfaces such as sandstone, shale, and kaolinite. This behavior was attributed to the unique chemical structure of the SDS/β-CD complex, which can self-assemble and release the surfactant before re-establishing the complex rapidly through weak van der Waals interactions. The CD cavity can encapsulate the surfactant and provide a protective effect, thereby preventing surfactant adsorption onto the rock surface. It should be noted that both the porous medium (sandstone) and the surfactant (SDS) used in this study have a negative charge, which promotes electrostatic repulsion and naturally reduces surfactant adsorption on the rock surface. In the oil-saturated region of the reservoir, the CD cavity must bind to oil molecules due to its strong hydrophobicity so that the encapsulated surfactant can leave the β-CD cavity and enter the formation pores to perform its functions.^169,170,315^ However, this molecular arrangement designed for the delivery of anionic surfactants is unsuitable for application in carbonate rocks. In this context, the electrostatic interaction responsible for surfactant adsorption on the surface can be more intense than the hydrophobic interactions required to retain the surfactant in the CD cavity.

More recently, Romero-Zerón et al. employed an encapsulated surfactant system (ESS) to inhibit surfactant adsorption on solid surfaces and evaluated surfactant release by analyzing dynamic adsorption and quartz crystal microbalance. This study highlighted the importance of the encapsulator (carrier) in the EOR system. The authors observed that surfactant encapsulation effectively prevents surfactant loss due to adsorption onto solid surfaces and maintains the integrity of the surfactant mass flowing through sand-based porous media systems.^317^ The CD cavity exhibits the capability to release surfactant molecules and encapsulate oil molecules owing to its pronounced hydrophobic nature. Consequently, the encapsulated surfactant is liberated from the cavity, allowing it to penetrate the pores of the formation and perform its designated functions.^316^

The research conducted by Luis A. Alcázar-Vara and collaborators focuses on utilizing a surfactants encapsulated in supramolecular complex formed by the interaction between cocoamidopropyl hydroxysultaine, sodium dodecyl alpha-olefin sulfonate, and sodium dodecyl hydroxyl sulfonate. This complex is designed to manage and regulate asphaltene deposition on rock surfaces, thereby safeguarding the integrity of the formation, while also reducing the concentration of heavy oils through solubilization. Although the study does not specifically examine the behavior of this supramolecular system in the presence of surfactants, it provides a foundation for potential evaluation as a surfactant carrier.^318^

CD molecules can be added to the polymer structure in β-CD polymer flooding due to their associations with hydrophobic groups, strong steric hindrance, and good rigidity. Introducing hydrophobic side groups can enhance polymers’ temperature and salt tolerance, and their thickening ability and strong viscoelasticity can improve the reservoir flow resistance.^319,320^

In a binary system comprising surfactant and polymer, there is an increase in surfactant storage. Polymers can bind surfactant molecules through the inclusion associations of CD cavities on the molecular chain or through a combination with ionic surfactants. This can improve the physicochemical and rheological properties of the original system. Another advantage is the synergistic effect of this series of polymers with CD side groups.^321−326^ An ideal model of synergistic action between CD-polymer and surfactant flooding is shown in Figure 9. The surfactant binds to the polymer through CD cavities. On the other hand, polymer-surfactant interactions can also occur through other parts of the polymer structure due to hydrophobic associations, thus improving the storage capacity.^321−326^

The main characteristics of nanocomposites of β-CD-functionalized polymer for flooding are high rigidity, thermal stability, and salt and shear resistance.^41^ This can be attributed to the hydrogen bonds between the bulk polymer and the nanoparticles, which make the polymer network structure denser and stronger, providing steric hindrance and inhibiting the coiling of polymer chains at elevated temperatures. Another advantage is the resistance factor (RF) and the residual resistance factor (RRF), indicating the nanocomposite’s potential for EOR. Other works have shown the use of nanoparticles as rock wettability modifiers and nanocarriers, as discussed in Section 3.1. Thus, these systems can also act as surfactant delivery for the EOR process.

The supramolecular class of surfactant delivery systems was pioneered by cyclodextrin, which forms a guest–host complex between the surfactant tail and the cyclodextrin cavity. However, its low surfactant storage capacity, limited to a single molecule per cyclodextrin molecule, remains a significant drawback. To increase storage capacity, researchers have proposed derivatives of cyclodextrin-containing active sites and incorporated them into the structure of polymers or nanocomposites. Both linear and hyper-branched polymers have been chosen for modification with cyclodextrin derivatives. These modified systems have been shown to be more efficient than their isolated counterparts, as they reduce surfactant adsorption on the rock and transport a greater number of surfactants.

Polymers and supramolecular systems are highly efficient surfactant carriers with different approaches as summarized in Table 4. They have high solubility and small sizes, decreasing the possibility of pores obstruction in the reservoir. They show high surfactant storage capacity and can act as controlled delivery surfactant systems due to intra and intermolecular interactions. They reduce interfacial tension to ultralow values and act on fluid viscosity, reducing capillary forces in the EOR process. However, synthesizing these systems is complex, decreasing their economical viability.

**Table 4:** Surfactant Carriers Based on Polymers and Supramolecular Systems

Type	Carrier	Surfactant	Mechanism	Advantage	Disadvantage	Ref
Supra-molecular	CD	SDS	host–guest complexes	Specificity	Delivery only one molecule	(169), (170), (315)
Supra-molecular	CD-polymer	SDS	host–guest complexes	High surfactant loading, thermal stability, and salt and shear resistance	Complex synthetic route	(316)
Polymer	Chitosan derivatives	nonionic surfactant NF-10 (nonylphenol), SDS, DEA	ultralow IFT	High solubility and surfactant loading	precipitates in high pressure	(282), (310), (311), (313)
Polymer	HPG	cationic surfactants	ultralow IFT	High surfactant loading, thermal stability, salt resistance	Complex synthetic route	(172)
Organic NP	Polystyrene NP	NF-10	Surfactant delivery by particle swelling	Controlled surfactant release	Low stability in brine	(35)
Organic NP	Sulfonated Polystyrene NP	OADA	Surfactant delivery by particle swelling.	Low IFT. Controlled surfactant release	Aggregation, low salt resistance	(37)
Organic NP	Lipidic matrix	NF-10	Particle dissolution	ultralow IFT to	Low thermal stability and salt resistance	(39)

## 4 Conclusions and Future Perspectives

Surfactant carriers play a crucial role in transporting surfactants through porous media and delivering them to the water–oil interface while minimizing surfactant loss due to adsorption on rock surfaces. These carriers can also improve interfacial properties in the reservoir and increase oil recovery factors.

In the context of the current global oil production scenario and EOR methods, developing new EOR technologies is motivated by the low recovery factor. Understanding the fundamental concepts and properties of EOR is essential for defining appropriate and economically viable strategies to improve the efficiency of surfactant flooding.

Selecting the appropriate surfactant carrier for specific reservoir and crude oil conditions presents a significant challenge due to the multitude of potential applications and action mechanisms involved. Nevertheless, given that the primary drawbacks of polymeric surfactants or supramolecular systems are often linked to synthetic routes, a surfactant carrier derived from these systems may prove to be the most suitable option, provided it meets the minimum requirements for reducing adsorption through chemical interactions.

For instance, if oil viscosity significantly impacts oil recovery in a particular reservoir, opting for a polymer or polymeric surfactant as a carrier can mitigate mobility ratio issues, thereby enhancing sweep efficiency. This approach may yield a synergistic effect, increasing oil recovery factors.

Conversely, in cases where wettability poses challenges in depleted reservoirs, employing inorganic nanoparticles or carbon materials alongside surfactants can help alter the rock’s wettability. Additionally, the adsorption of these nanoparticles onto the rock surface can block isolated pores, thereby improving the direction of oil flow. It is important to note that creating various scenarios for the application of the surfactant carrier is beyond the scope of this review. The ultimate decision in this regard should be made considering factors of reservoir engineering beyond the efficiency and action mechanisms of the chemical additives. However, the perspectives discussed herein are considered valuable insights that can aid in making an informed choice.

Although surfactant carrier systems have high potential applications in EOR, few studies have shown performance tests such as coreflooding and imbibition. The main future perspectives are evaluating performance tests in reservoir conditions, such as high temperature, pressure, and salinity. The economic aspect of carrier production and efficiency should also be evaluated. Carbon materials, biopolymers, and iron or silicon oxide nanoparticles have high potential as surfactant carriers for EOR, and one can expect to see an increase in their use in the EOR process in the near future.
